# Atlastin-1 regulates morphology and function of endoplasmic reticulum in dendrites

**DOI:** 10.1038/s41467-019-08478-6

**Published:** 2019-02-04

**Authors:** Xianzhuang Liu, Xiangyang Guo, Liling Niu, Xixia Li, Fei Sun, Junjie Hu, Xiangming Wang, Kang Shen

**Affiliations:** 1 National Laboratory of Biomacromolecules, Institute of Biophysics, Chinese Academy of Sciences, 15 Datun Road, Chaoyang District, Beijing, 100101 China; 2 University of Chinese Academy of Sciences, Beijing, 100101 China; 3 Department of Genetics and Cell Biology, College of Life Sciences, Nankai University, Tianjin, 300071 China; 4 Center for Biological Imaging, Institute of Biophysics, Chinese Academy of Sciences, 15 Datun Road, Chaoyang District, Beijing, 100101 China; 5 Howard Hughes Medical Institute, Department of Biology, Stanford University, Stanford, California, USA

## Abstract

Endoplasmic reticulum (ER) is characterized by interconnected tubules and sheets. Neuronal ER adopts specific morphology in axons, dendrites and soma. Here we study mechanisms underlying ER morphogenesis in a *C. elegans* sensory neuron PVD. In PVD soma and dendrite branch points, ER tubules connect to form networks. ER tubules fill primary dendrites but only extend to some but not all dendritic branches. We find that the Atlastin-1 ortholog, *atln-1* is required for neuronal ER morphology. In *atln-1* mutants with impaired GTPase activity, ER networks in soma and dendrite branch points are reduced and replaced by tubules, and ER tubules retracted from high-order dendritic branches, causing destabilized microtubule in these branches. The abnormal ER morphology likely causes defects in mitochondria fission at dendritic branch points. Mutant alleles of Atlastin-1 found in Hereditary Spastic Paraplegia (HSP) patients show similar ER phenotypes, suggesting that neuronal ER impairment contributes to HSP disease pathogenesis.

## Abstract (web summary)

The molecular mechanisms that achieve ER morphology in neurites are not well understood. This study uses a forward genetic approach to demonstrate that *atln-1* is required for neuronal ER morphology and that *C. elegans atln-1* mutants exhibit defects in mitochondria fission at dendritic branch points and abnormalities in protein homeostasis.

## Introduction

The endoplasmic reticulum (ER) forms a continuous intracellular membrane system, consisting of the nuclear envelope, flat sheets, and elaborate network of tubules throughout cytoplasm^1,2^. ER functions in a number of essential cellular processes such as synthesis of transmembrane and secreted proteins, post-translational modification of proteins, lipid synthesis, and Ca^2+^ homeostasis^3^.

The highly complex and dynamic morphology of ER is important for its functions. A number of proteins have been implicated in the morphogenesis of ER^4,5^. The integral membrane protein Rtn4a/NogoA, a member of the ubiquitously expressed Reticulon family, functions in generating tubular ER^6^. Reticulon interacts with DP1/Yop1p, a conserved integral ER membrane protein to stabilize ER tubules by maintaining membrane curvature^6^. The ER localized dynamin-like GTPase Atlastin-1 (ATL) promotes the fusion between tubules to form ER networks^7,8^. ATL is highly conserved during evolution with homologs in yeast (Sey1) and plants (RHD3)^9^. Additional proteins including Climp-63, Kinectin, p180, Lunapark, and Protrudin have also been implicated in establishment and maintenance of ER morphology^10–12^.

Differentiated cells adopt cell type specific ER morphology. For example, muscle cells use the specialized form of ER, sarcoplasmic reticulum (SR), to achieve tight coupling of membrane depolarization and contraction^13^. In yeast and plant cells, a large portion of the ER is concentrated underneath the plasma membrane^14^. In neurons, ER contains mainly tubular form in axons^15^, and both networks and tubules in dendrites^16,17^. ER complexity within dendrites increases at branch points in mammalian neurons^18^. The molecular mechanisms that generate and maintain the specific ER morphology in the dendrites are not fully understood.

Impairment of ER function might be related to many human diseases such as Hereditary Spastic Paraplegia (HSP). HSP is a neurological disorder with progressive spasticity and weakness of the lower extremities^19^. More than 50 genetic loci have been found to be genetic causes of HSP^20^. Interestingly, many mutations in Atlastin-1 are found in HSP patients, suggesting that ER morphology might be affected in neurons of HSP patients^21^. Indeed, a series of studies have examined the function of Atlastin in axonal ER. In *Drosophila* motor neurons, downregulation or overexpression of Altastin cause reduced spontaneous release and the reserved pool of synaptic vesicles^22^. In another study, in Altastin mutants, ER networks in the *Drosophila* motor axon terminal become fragmented^23^. While the majority of the literature has focused on Altastin’s function in the axonal ER, several papers also examine its potential function in dendrites. In one study, overexpression of a GTPase dead form of Atlastin-1 in neurons leads to a decrease of ER in dendritic spines and reduced protein synthesis^24^. In another study, overexpression of wild type but not a GTPase defective form of Atlastin-1 in cortical neurons causes increased growth and branching of dendrites, suggestive of a function in dendrite morphogenesis. However, the relationship between neuronal ER impairment, Atlastin-1 and HSP has not been directly established.

The PVD neuron of *C. elegans* is a sensory neuron with elaborate dendritic arbors. The PVD dendrite consists of stereotyped, orthogonally oriented primary, secondary, tertiary, and quaternary branches^25,26^. In this study, we use the PVD neuron as a model to study ER morphology and function in dendrites. Using a forward genetic screen, we isolated *atln-1* mutants, which showed reduced ER tubules in the dendrite branches. We found that *atln-1* mutant had impaired GTPase activity and was unable to fuse membrane both in vivo and in vitro. We also report that the abnormal ER morphology led to defects in mitochondria attachment and fission at dendritic branch points and abnormalities in protein homeostasis when the unfolded protein response (UPR) was compromised. Mutant alleles of Atlastin-1 found in HSP patients showed similar ER defects, suggesting that neuronal ER impairment contributes to HSP pathogenesis.

## Results

### ATLN-1 is required for ER tubule extension into dendrites

**Fig. 1:**
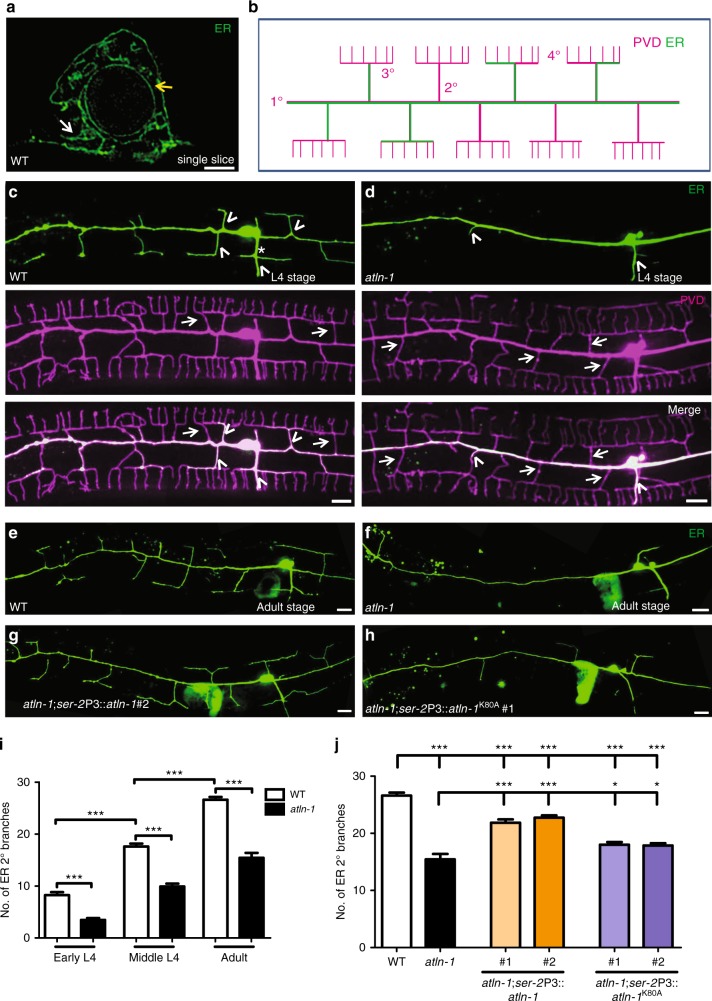
ATLN-1 is required for ER invasion into dendrites. **a** A representative 3D-SIM image of ER (GFP::SP12) in wild-type PVD soma (A single focal plane is shown). The yellow arrow indicates the nuclear membrane and the white arrow indicates a tubular network. Scale bar, 1 μm. **b** A schematic picture showing that ER extends into some but not all 2° and 3°dendrites in PVD. Magenta lines represent PVD dendritic branches and green lines indicate ER distribution. **c**, **d** Representative confocal images of ER (GFP::SP12) and dendritic morphology of PVD neuron (*ser-2*P3::mCherry) in wild type (**c**) and *atln-1*(*wy50080*) mutant (**d**) at L4 stage. Arrows show PVD 2° branches without ER and arrowheads indicate PVD 2° branches with ER. Asterisks mark the PVD axon. Scale bars, 10 μm. **e–h** Representative ER morphology of PVD neuron in WT (**e**), *atln-1* (**f**)*, atln-1; ser-2*Prom3::*atln-1* #2 (transgenic line #2) (**g**) and *atln-1; ser-2*Prom3::*atln-1* ^K80A^ #1(transgenic line #1) (**h**) at adult stage. Scale bars, 10 μm. **i**, **j** Quantification of ER 2° branches number. Values are mean and error bars are SEM, *n* = 46 for each genotype. *p < 0.05; ***p < 0.001 (two-sided student’s *t* test and Tukey’s multiple comparison test)

To visualize the ER morphology in the PVD neuron, we expressed the ER residential protein: signal peptidase (SP12)^27^ fused with GFP with a PVD specific promoter, *ser-2*Prom3^28–30^. Using Three-Dimensional Structured Illumination Microscopy (3D-SIM), we observed that the GFP fluorescence pattern in the PVD soma exhibited a typical ER pattern, including a nuclear envelope and sparse connected tubule networks (Fig. 1a). Consistent with previous reports, ER extended to both axon and dendrites (Fig. 1b, c). Surprisingly, within dendrites, ER was only found in a subset of dendritic branches. ER filled the entire length of the primary dendrites, but only extended to a fraction of the secondary (2°) and tertiary (3°) dendrites, and very rarely extended to tertiary (4°) dendrites (Fig. 1b, c). The number of 2° branches containing ER gradually increased after the entire dendritic branches formed at L4 stage, indicating that the ER extension to the dendritic branches significantly trailed dendrites formation (Fig. 1i). To validate the SP12 marker, we also expressed several other ER proteins such as ESYT-2 and SEC-61 fused with GFP in PVD. They both showed similar patterns as GFP::SP12 in both the PVD soma and dendrites (Supplementary Fig. 1E, G and J).

To understand the molecular mechanisms underlying ER distribution in dendrites, we carried out an unbiased, forward genetic screen using this ER marker. From about 4000 mutated haploid genomes, we isolated 12 mutants with abnormal ER pattern. Among them, the *wy50080* mutant showed dramatically reduced ER tubules in 2° and 3° dendrites at all developmental stages compared to the wild type, regardless of the ER markers (Fig. 1c–f, i; Supplementary Fig. 1E–I). Using SNP mapping and sequencing, we identified the causative mutation in the *atln-1* gene, the ortholog of the vertebrate Atlastin-1 (Supplementary Fig. 1A). PVD specific expression of *atln-1* rescued the ER phenotype of *wy50080* (Fig. 1g, j), indicating that *atln-1* functioned cell-autonomously in PVD to regulate ER morphology.

In non-neuronal cells, Atlastin-1 is an ER localized GTPase that establishes ER networks by mediating homotypic membrane fusion^7,8^. To test if the GTPase activity of ATLN-1 was essential for its function in ER morphology in PVD, we introduced the K80A mutation into *altn-1*, a mutation that disrupts GTP binding in the vertebrate Atlastin-1^31^. The ATLN-1^K80A^ transgene failed to fully rescue the ER phenotype in the *atln-1* mutants (Fig. 1h, j), indicating that the GTPase activity of ATLN-1 is critical for dendritic ER morphology. The subtle rescue activity might be due to the partial allelic complementation between the two mutant alleles.

**Fig. 2:**
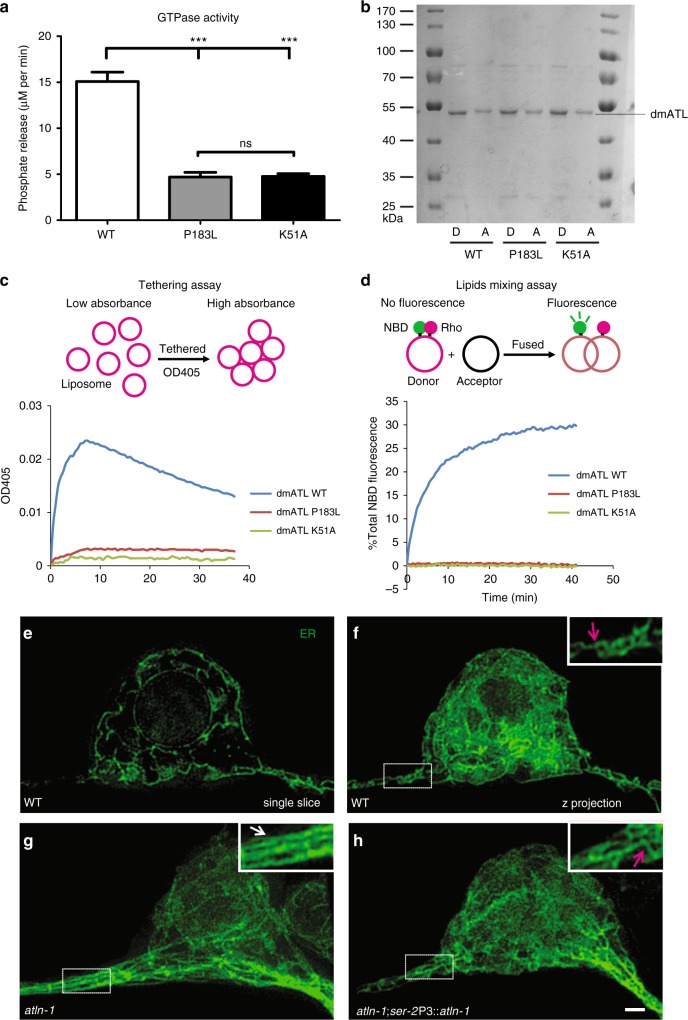
ATLN-1 regulates somatic ER network formation. **a** Quantification of GTPase activity of WT and mutant dmATLs. The activities were measured by phosphate release at saturating GTP concentrations of 0.5 mM. At least three repetitions were performed for each group. Values are mean and error bars are SEM. ns, not significant; ***p < 0.001 (Tukey’s multiple comparison test). **b** Reconstitution of *Drosophila* WT, P183L, and K51A dmATL proteins into liposomes. Donor (**D**) and acceptor (**A**) vesicles with recombinant proteins were analyzed by SDS/PAGE and Coomassie blue staining. (**c**) Schematic illustration of liposome membrane tethering assay (upper panel). The clustering of *Drosophila* WT and mutant dmATL containing proteoliposomes (protein:lipid ratio 1:2,000) was measured by the light absorbance at a wavelength of 405 nm (lower panel). **d** Schematic illustration of lipid mixing assay (upper panel). *Drosophila* WT and mutation dmATL were reconstituted at equal concentrations into donor and acceptor vesicles. GTP-dependent fusion of donor and acceptor vesicles was monitored by the dequenching of an NBD-labeled lipid in the donor vesicles (lower panel). Fusion was initiated by addition of GTP. **e** Representative 3D-SIM image of PVD somatic ER in a WT worm (single focal plane). **f**–**h** Representative 3D-SIM maximum-intensity-projection images of PVD somatic ER in WT (**f**), *atln-1* (**g**) and *atln-1; ser-2*P3::*atln-1* worms at adult stage. Insets show 2X magnified views. The magenta arrows indicate three-way junction and white arrows indicate parallel ER tubules. Scale bar, 1 μm

The mutation in *atln-1(wy50080*) is a missense mutation that causes the amino acid substitution P219L, which localizes in the GTPase domain. We used in vitro assays to test whether this mutation affected the GTPase activity as well as the membrane tethering and fusion activity. We introduced the corresponding mutation P183L in the *Drosophila* ortholog dmATL and used a previously characterized K51A mutation as a positive control. The K51A mutation was shown to reduce GTPase activity and completely lack tethering and fusion activity^8,32^. Interestingly, the GTPase activity of P183L mutant was drastically reduced, similar to that of K51A (Fig. 2a). Wild-type and mutant dmATL were then purified and reconstituted into vesicles for membrane tethering and fusion activity analyses (Fig. 2b). Tethered vesicles caused an increase in turbidity, which could be monitored by measuring light absorbance at 405 nm (Fig. 2c). A fluorescence de-quenching assay was used to determine fusion activity based on lipid mixing. The donor vesicles contained lipids labeled with both nitrobenzoxadiazole (NBD) and rhodamine at quenching concentrations. Once fused with acceptor vesicles, the labeled lipids became diluted, resulting in increase in NBD fluorescence (Fig. 2d). Similar to K51A, P183L mutant completely lacked membrane tethering and fusion activity (Fig. 2c, d). Thus, P183L behaved indistinguishably as K51A in all three assays, indicating that the *atln-1* mutant (P219L) should have dramatically reduced GTPase activity and might have defects in ER tubule fusion and networks formation.

Atlastin-1 functions as dimer when mediating homotypic ER membrane fusion^7^. Most Atlastin-1 mutations found in HSP patients are heterozygous mutations that function as dominant negative alleles to further inhibit the function of the wild-type allele. To distinguish whether the P219L allele was a loss-of-function or dominant negative allele, we overexpressed the mutant P208L (P208L is the equivalent mutation in the mammalian ATL1) as well as the known K80A ATL1 in wild-type COS-7 cells, which endogenously express the ATL2 and ATL3 paralogs but not ATL1 itself. As expected, the K80A ATL1 caused a reduction in the density of 3-way junctions of the ER networks due to its dominant negative effects. The P208L ATL1 did not show a similar detrimental effect (Supplementary Fig. 2A). We further tested if P208L was a loss-of-function mutant with rescue experiments. COS-7 cell lines lacking both ATL2 and ATL3 showed reduced density of ER 3-way junctions. This phenotype could be rescued by wild-type ATL1 but not the P208L or K80A forms, further confirming that the P208L allele represented a loss-of-function allele (Supplementary Fig. 2B). To test if the worm P219L *atln-1* behaved similarly in PVD, we expressed the *atln-1*(P219L) in wild-type PVD and observed no dominant negative effect (Supplementary Fig. 2F–H). Furthermore, we did not observe any ER phenotype in the *atln-1* mutant (P219L) heterozygotes (Supplementary Fig. 2E, H). Together, these data indicate that P219L is a loss-of-function allele.

To directly test if ATLN-1 was required for ER membrane fusion in neurons, we imaged the ER networks in the PVD soma using 3D-SIM. In wild-type controls, ER formed complex networks characterized by numerous three-way junctions (Fig. 2e, f). While in *atln-1* mutants, the ER networks were visibly less complex with fewer three-way junctions and dramatically increased parallel, unbranched ER tubules (Fig. 2g). This ER morphology defect was rescued by a wild-type ATLN-1 transgene expressed in PVD (Fig. 2h). Both the reduction of ER networks and the appearance of parallel tubules in the *altn-1* mutant were strikingly similar to the ER defects in mammalian non-neuronal cells lacking Atlastin-1^7^. Consistent with its ER shaping function, we found that GFP::ATLN-1 was localized to numerous tubule structures in the cell body and tubules in the dendrites that highly resembled the ER pattern in the PVD soma and dendrites (Supplementary Fig. 1B–D). Together, these evidences suggest that ATLN-1 is localized on neuronal ER and is required to establish somatic ER tubule networks in PVD.

### *atln-1* mutant causes excessive ER retraction in PVD

**Fig. 3:**
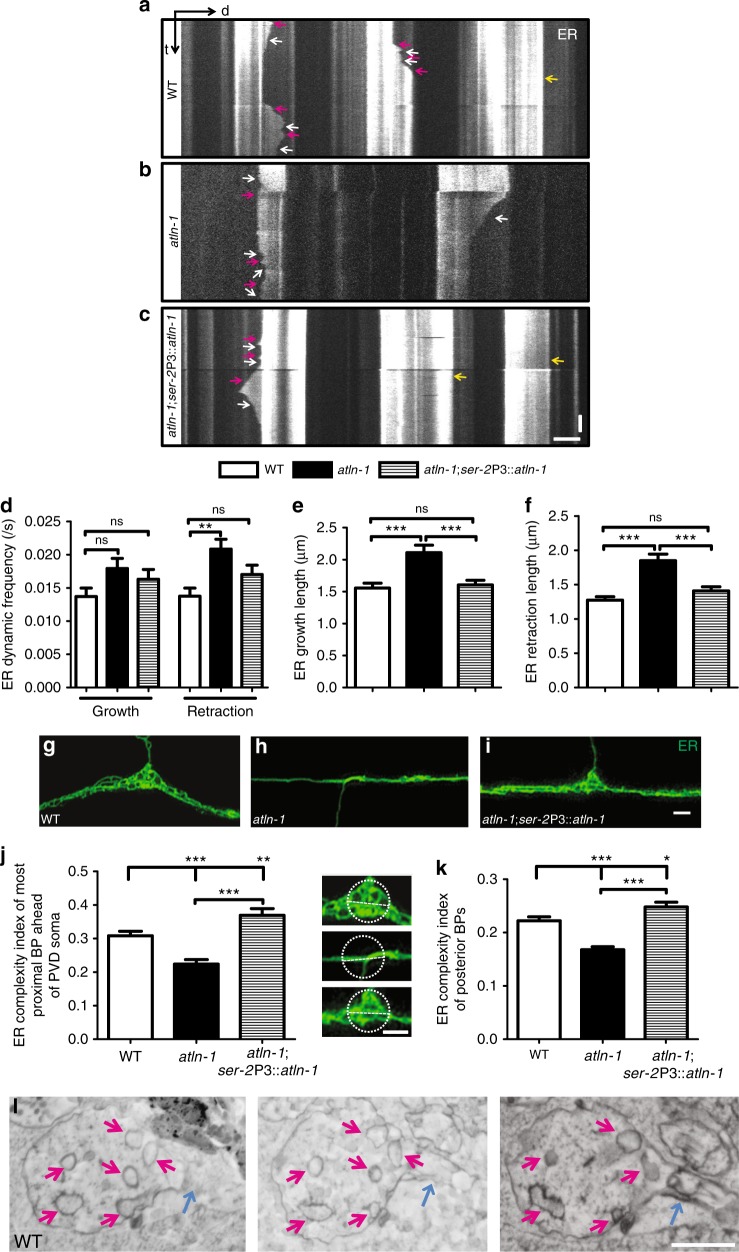
a*tln-1* mutants show high level of ER retraction. **a**–**c** Kymographs of ER (GFP::SP12) dynamic events in PVD 3° branches in WT (**a**), *atln-1* (**b**), and *atln-1; ser-2*P3::*atln-1* (**c**) worms at L4 stage. Magenta, white and yellow arrows indicate ER growth, retraction, and static events, respectively. Scale bar, 5 μm and 10 s. **d** Quantification of ER growth and retraction frequency. Values are mean and error bars are SEM. At least 200 events from more than 30 worms are quantified. ns, not significant, **p < 0.01 (Newman-Keuls multiple comparison test). **e,** **f** Quantification of ER growth (**e**) and retraction (**f**) length. Values are mean and error bars are SEM. At least 200 events from more than 30 worms are quantified. ns, not significant; ***p < 0.001 (Newman-Keuls multiple comparison test). **g**–**i** Representative 3D-SIM images of ER (GFP::SP12) complex structure at the most proximal branch point of the PVD anterior dendrite in WT (**g**), *atln-1* (**h**), and *atln-1; ser-2*P3::*atln-1* (**i**) worms. **j** Quantification of ER complexity index of the most proximal branching point. Values are mean and error bars are SEM, *n* > 40. **p < 0.01; ***p < 0.001 (Newman-Keuls multiple comparison test). To calculate the complexity index, we drew a circle of 1.6 μm diameter (dashed circle) at the branching point and a line within the circle along the dendrite trunk (dashed line). Complexity index = mean GFP intensity in the circle/mean GFP intensity on the line. Scale bars, 1 μm. **k** Quantification of ER complexity index at posterior branch points. Values are mean and error bars are SEM. At least 100 branch points from more than 30 worms are quantified. *p < 0.05; ***p < 0.001 (Newman-Keuls multiple comparison test). **l** Scanning electron microscopy images from a serial EM pictures showing ER complex structure on a branch point in PVD of a WT worm. The magenta arrows indicate ER. The blue arrows indicate PVD 2° branches. Scale bar, 500 nm

To understand why ER branches were reduced in PVD dendrites in the *atln-1* mutants, we performed time-lapse imaging experiments to record the ER (GFP::SP12) dynamic in PVD tertiary dendrites. In wild-type worms, some ER tubules showed a dynamic pattern characterized by growth and retraction, while others were stable during the imaging experiments (Fig. 3a). In *atln-1* mutants, we found that the frequency of ER extension into the branches was not changed, but the retraction frequency was increased when compared with wild-type worms (Fig. 3a, b, d). PVD specific expression of ATLN-1 could fully rescue this phenotype (Fig. 3c, d). Also, we found that both the growth and retraction length were increased, which was fully rescued by PVD specific expression of *atln-1* (Fig. 3e, f). These results suggest that the reduction of ER in dendritic branches in *atln-1* mutants might be caused by excessive retraction.

Next, we investigated why ER tubules retracted from dendrite branches in *atln-1* mutants. The ER tubules in branches originated from tubules in the primary dendrite. We noticed that ER formed small network structures at the branch sites where the volume of the neurites were greater compared with the dendritic trunk (Fig. 3g). Using “automatic collector of ultrathin sections scanning electron microscopy” (AutoCUTS-SEM)^33^, we reconstructed a segment of the PVD primary dendrite containing a 2° branch site. The ultrastructure showed that there were multiple ER cross section-like profiles at the branch site, consistent with a small ER network (Fig. 3l). Using the super-resolution SIM imaging, we found that the ER networks at the branch points were replaced by unbranched tubules in *atln-1* mutants, which was rescued by PVD expression of *atln-1* (Fig. 3h–k), indicating a key role of *atln-1* in formation of ER networks at branch points. Since ER tubules in 2° dendrites originated from the ER networks at branch points, we speculated that ER networks might promote ER tubules formation or stabilization in 2° dendrites. To test this hypothesis, we divided branch points (BPs) into two types: the ones with ER tubules extending into the corresponding 2° dendrites (+ER BPs) and the ones without ER tubules in the 2° dendrites (−ER BPs). We then compared the ER complexity at these two types of BPs. More complex ER networks were found in the +ER BPs compared to the −ER BPs (Supplementary Fig. 3A–C), which is consistent with the notion that ER networks at BPs may help to stabilize ER tubules in the 2° dendrites.

### ER and microtubules (MTs) mutually affect each other in dendrites

In non-neuronal cells, ER tubules extend along MTs using sliding mechanism or tip attachment complex (TAC) mechanism^34^. To investigate if the ER extension defect in *atln-1* mutants was related with MTs, we expressed the α-tubulin protein TBA-1 fused with GFP to monitor MT’s pattern and dynamics in PVD^35^. Interestingly, MTs showed very similar pattern as ER in PVD dendrites: both ER and MTs were present in a fraction of secondary and tertiary dendrites (Supplementary Fig. 4B). Our unpublished results showed that ER extension into the PVD 2° and 3° branches was strictly dependent on the transient presence of MTs during the ER outgrowth events, while retraction of ER from the branches was not (unpublished results X. L., X. W. and K. S.). Further, co-localization analyses showed that ER and MTs were mostly present in the same branches, and *atln-1* mutants did not change the co-localization of ER and MTs (Supplementary Fig. 4A–D), consistent with the notion that ER might enter the branches through a MTs dependent mechanism.

**Fig. 4:**
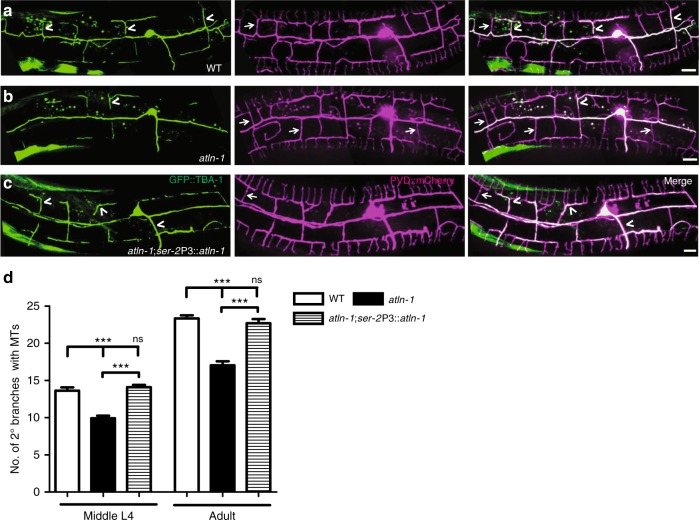
ATLN-1 affects the presence of MT in dendritic branches. **a**–**c** Representative confocal images of MTs (GFP::TBA-1) and dendritic morphology of PVD neuron (*ser-2*P3::mCherry) in WT (**a**), *atln-1* (**b**), and *atln-1; ser-2*Prom3::*atln-1* adult worms (**c**). Arrows show PVD 2° branches without MTs and arrowheads indicate PVD 2° branches with MTs. Scale bars, 10 μm. **d** Quantification of the number of 2° branches with MTs. Values are mean and error bars are SEM, *n* > 40. ns, not significant; ***p < 0.001 (Newman-Keuls multiple comparison test)

We also noticed that MTs (GFP::TBA-1) within the 2° and 3° branches were significantly reduced in the *atln-1* mutants compared with wild-type controls (Fig. 4a, b, d). This phenotype was found in both L4 and adult worms and could be largely rescued by PVD expression of wild-type *atln-1*, suggesting that ATLN-1 affects MTs distribution in dendritic branches (Fig. 4c, d).

**Fig. 5:**
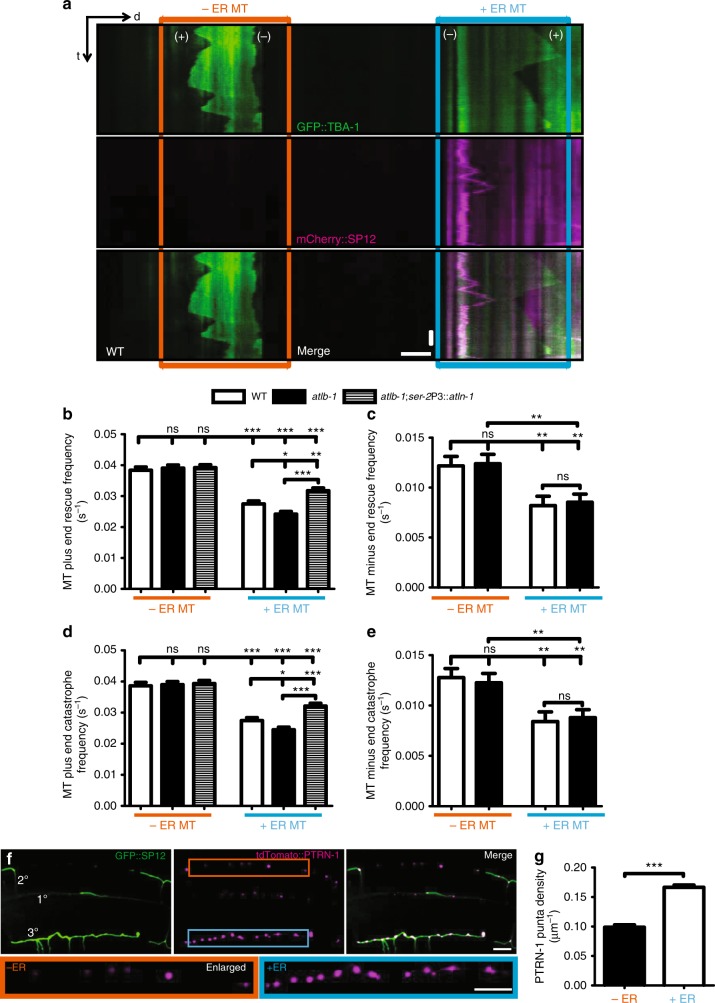
MT dynamics and numbers are affected by the ER. **a** Kymographs recording the dynamic events of MT (GFP::TBA-1) and ER (mCherry::SP12) in 3° branches from a L4 stage WT animal. A blue box labels a +ER MT, while an orange box labels a –ER MT. MT’s plus end is marked by (+) and minus end is marked by (−). Scale bar, 5 μm and 15 s. **b**–**e** Quantification of dynamic parameters of MT plus ends (**b**, **d**) and minus ends (**c**, **e**). The frequency of rescue (**b**, **c**) and catastrophe (**d**, **e**) were plotted. Values are mean and error bars are SEM. At least 150 events from more than 30 worms are quantified. ns, not significant; *p < 0.05; **p < 0.01; ***p < 0.001 (Newman-Keuls multiple comparison test). **f** Confocal images showing tdTomato::PTRN-1 puncta in PVD 3° branches at adult stage. +ER (in blue box) means a 3° dendrite with ER present. -ER (in orange box) means the 3° branch without ER present. Scale bars, 10 μm. **g** Quantification of PTRN-1 puncta density in −ER and +ER 3° dendrites. Values are mean and error bars are SEM. At least 100 confocal views of PVD proximal regions from more than 40 worms are quantified. ***p < 0.001 (two-sided student’s *t* test)

To further understand ATLN-1’s role in MT regulation, we conducted time-lapse imaging experiments to study the MTs’ dynamic behavior in tertiary branches. MTs were sparsely distributed in 3° branches, providing us with the resolution to visualize single MT filament^36,37^. From kymograph analyses, we could often observe the distinct dynamic behaviors of two ends of the same MTs (TBA-1::GFP, Fig. 5a). We designated the more dynamic ends as the plus-ends and the more stable ends as minus-ends (Fig. 5a). To understand the relationship between ER and MTs, we further divided MTs into two categories: MTs that overlap with an ER tubule (+ER) or do not overlap with ER (−ER) in the 3° branches (Fig. 5a).

In wild-type animals, we found that the growing ends of MTs were less dynamic for +ER MTs compared with the −ER MTs. This was reflected by decreased frequency of both MTs catastrophe and rescue in the +ER MTs (Fig. 5a–e). *C. elegans* neurites contain partially tiled MT arrays. The number of MTs can be estimated by the density of minus-end marking PTRN-1 puncta along the neurites^37,38^. We found that the density of PTRN-1 puncta was higher in the +ER 3° branches compared to that of the –ER 3° branches (Fig. 5f, g). The +ER and –ER branches are indistinguishable from each other in morphology, and are both dependent on the same molecular pathways for their development^29,39^. These observations suggest that ER might stabilize nearby MTs in dendritic branches.

Since Atlastin has been shown to recruit MT severing enzyme spastin^40^, we next asked if MT dynamics were changed in the *atln-1* mutants. For the –ER MTs, we did not detect any difference between WT and *atln-1* mutants, strongly suggesting that *atln-1* does not directly regulate MT dynamics in PVD. For the +ER MTs, we found that *altn-1* mutant MTs’ plus-ends were slightly more stable compared to WT, suggesting that ATLN-1’s effect on MTs might be through affecting ER structure and function. These phenotypes were fully rescued by expression of wild-type ATLN-1 in PVD (Fig. 5b, d). Together, these results suggest that ER could stabilize adjacent MTs.

### ATLN-1 modulates mitochondria behavior in dendrites

**Fig. 6:**
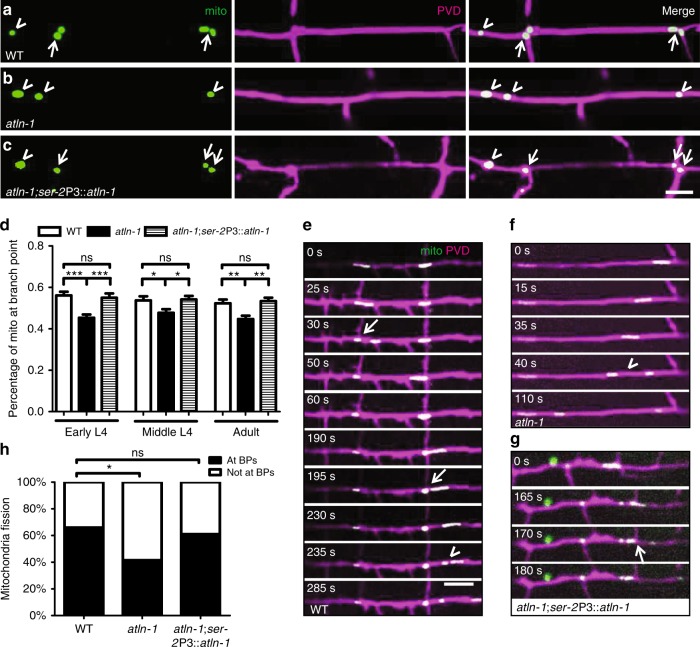
ATLN-1 modulates mitochondria dynamics in dendrites. **a**–**c** Mitochondria distribution in PVD primary dendrites in WT (**a**), *atln-1* (**b**) and *atln-1; ser-2*Prom3::*atln-1* (**c**) worms at adult stage. Arrows indicate mitochondria at branch points. Arrowheads indicate mitochondria which are not localized at branch points. Scale bar, 5 μm. **d** Quantification of the percentage of mitochondria localized at branch points. Values are mean and error bars are SEM, *n* > 25 for each genotype. ns, not significant; *p < 0.05; **p < 0.01; ***p < 0.001 (Newman-Keuls multiple comparison test). **e**–**g** Time-lapse images of mitochondria fission in WT (**e**), *atln-1* (**f**), and *atln-1; ser-2*P3::*atln-1* (**g**) worms. The arrows indicate mitochondria fission events at branch points. The arrowheads indicate mitochondria fission events outside of branch points. Scale bar, 10 μm. **h** Quantification of mitochondria fission events at and outside of branch points. *n* = 40–50. ns, not significant; *p < 0.05 (Chi-square test)

Above evidences showed that ALTN-1 was required for ER morphogenesis in the neuronal cell body and dendrites. Since ER is an expansive network and has been shown to contact other organelles and perform many cellular functions, we investigated if the ER morphology affected its functions. Interaction between ER and mitochondrion could enhance mitochondrion fission^41^. In neurons, ER-mitochondria contacts are critical for Ca^2+^ regulation^42^. To assess if the impairment of ER in *atln-1* mutant would affect mitochondria pattern in PVD dendrites, we marked mitochondria by expressing TOMM-20 (1-54aa)::GFP in PVD. We found that mitochondria were highly enriched at the dendritic branching sites: more than 50% of mitochondria in the primary dendrite were localized to the PVD dendritic branch sites at different developmental stages in wild-type worms (Fig. 6a, d). Since the branch sites contained ER networks, we hypothesized that mitochondria might be enriched there due to the local ER network.

To test this idea, we examined the mitochondria localization in the *atln-1* mutants where the branch sites localized ER networks were less complex. Compared with wild-type worms, the number of mitochondria localized to branch points was decreased at all the developmental stages in *atln-1* mutants (Fig. 6b, d). Since most mitochondria fission events occurred at ER-mitochondria contact sites^43^, we wondered if mitochondria fission might preferentially occurred at the branching sites and might be dependent on *atln-1*. Consistent with this hypothesis, mitochondria fission events at PVD dendritic branch sites were reduced from 66 to 41% in *atln-1* mutants (Fig. 6e, f, h). These phenotypes could be rescued by expression of wild-type ATLN-1 (Fig. 6c, d, g, h). In summary, these data suggest that local ER network at dendrite branch sites might anchor mitochondria and promote mitochondria fission.

### *atln-1* functions synergistically with *ire-1*

**Fig. 7:**
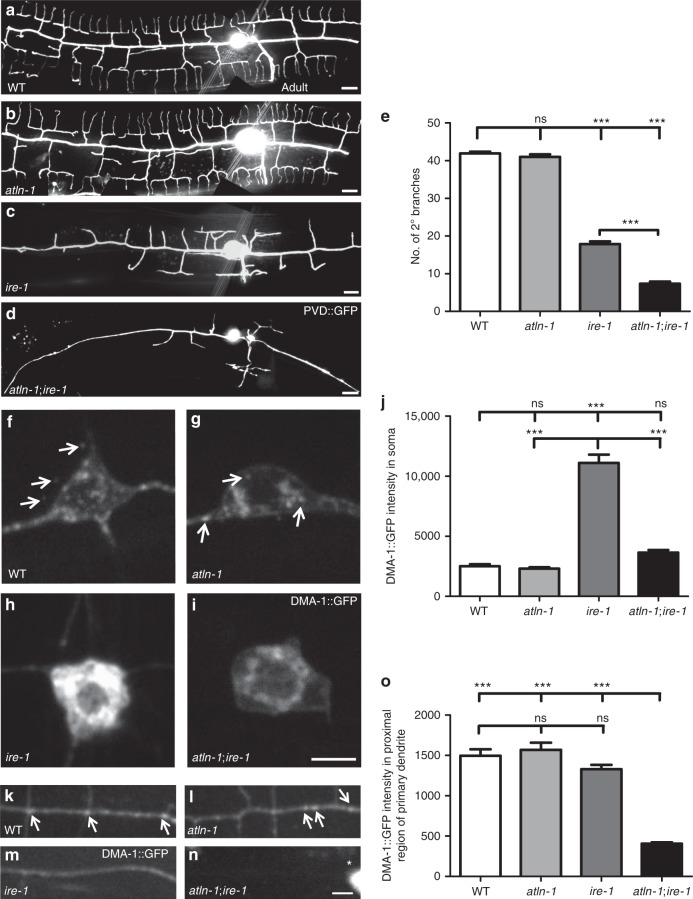
a*tln-1* functions synergistically with *ire-1*. **a**–**d** Representative confocal images of PVD (*ser-2*P3::GFP) morphology in WT (**a**), *atln-1* (**b**), *ire-1* (*ok799*) (**c**) and *atln-1; ire-1* (**d**) worms at adult stage. Scale bars, 10 μm. **e** Quantification of the number of PVD 2° branches. Values are mean and error bars are SEM, *n* > 40. ns, not significant; ***p < 0.001 (Newman-Keuls multiple comparison test). **f**–**i** Confocal images of DMA-1::GFP in PVD cell body in WT (**f**), *atln-1* (**g**), *ire-1* (**h**), and *atln-1; ire-1* (**i**) worms. Arrows indicate DMA-1::GFP puncta. Scale bar, 5 μm. **j** Quantification of DMA-1::GFP fluorescence intensity in PVD cell body. Values are mean and error bars are SEM, *n* = 40–50. ns, not significant; ***p < 0.001 (Newman-Keuls multiple comparison test). **k**–**n** Confocal images of DMA-1::GFP in anterior primary dendrite around the PVD soma in WT (**k**), *atln-1* (**l**), *ire-1* (**m**), and *atln-1; ire-1* (**n**) worms. Arrows indicate DMA-1::GFP puncta. Asterisk marks cell body. Scale bar, 5 μm. **o** Quantification of DMA-1::GFP fluorescence intensity of primary dendrites in (**k**–**n**). Values are mean and error bars are SEM, *n* = 40–50. ns, not significant; ***p < 0.001 (Tukey’s multiple comparison test)

The inositol-requiring enzyme 1 (IRE1) senses the ER stress and activates the UPR pathway^44^. Perturbation of the IRE1 arm of the UPR pathway causes loss of PVD dendritic branches^45^. We speculated that ER loss in PVD dendrites of *atln-1* mutant might further impair the ER function and block UPR. To test this idea, we constructed double mutants between *ire-1* and *atln-1*. Interestingly, the double mutants showed enhanced PVD dendrite arborization defect, although *atln-1* mutant alone did not show any visible dendrite morphogenesis phenotype (Fig. 7a–e).

We have previously shown that the UPR pathway is required to fold and deliver the dendrite arborization receptor DMA-1^45^. In wild-type animals, DMA-1::GFP was largely localized to secretory vesicle-like structures in the soma and showed  a diffuse plasma membrane-like pattern in the dendrites (Fig. 7f, k). In *ire-1* mutants, DMA-1 was largely trapped in the ER and reduced on the dendritic plasma membrane, especially at distal dendrites (Fig. 7f–h, j and Wei et al., 2015), leading to failed distal dendrites formation. Interestingly, in *ire-1*; *atln-1* double mutants, the overall level of DMA-1::GFP was greatly reduced compared with the *ire-1* single mutants (Fig. 7h–j). The dim DMA-1 signal still exhibited a predominant ER staining pattern in PVD soma (Fig. 7i). In the double mutants, even the proximal dendrites were nearly completely devoid of DMA-1 signal (Fig. 7k–o), which likely caused the enhanced dendrite arborization defect. These results suggest that ER networks morphology might affect homeostasis of neuronal membrane proteins and this requirement is particularly important when UPR is compromised.

### HSP disease alleles of Atlastin-1 affect ER morphology

**Fig. 8:**
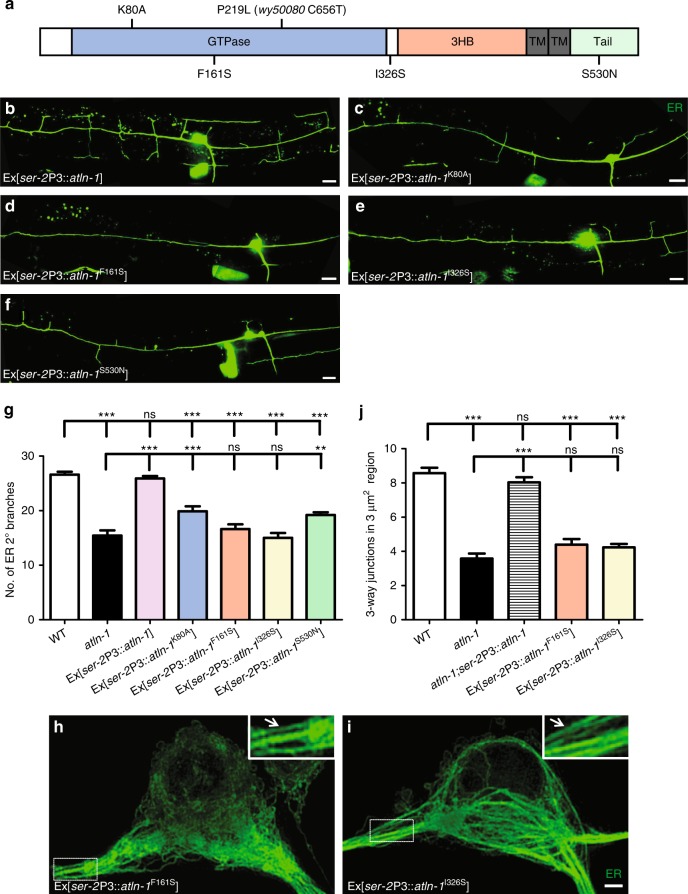
HSP disease alleles of Atlastin-1 affect ER morphology. **a** Schematic diagram of ATLN-1 protein domains. K80A, *wy50080* (P219L) and HSP related disease mutations are showed. **b**–**f** Representative ER morphology in Ex[*ser-2*P3::*atln-1*] (**b**), Ex[*ser-2*P3::*atln-1*^K80A^] (**c**), Ex[*ser-2*P3::*atln-1*^F161S^] (**d**), Ex[*ser-2*P3::*atln-1*^I326S^] (**e**) and Ex[*ser-2*P3::*atln-1*^S530N^] (**f**) transgenic adult worms. Scale bars, 10 μm. **g** Quantification of ER 2° branches number. Values are mean and error bars are SEM, *n* = 40–50. ns, not significant; **p < 0.01; ***p < 0.001 (Tukey’s multiple comparison test). **h**, **i** Representative maximum-intensity-projection 3D-SIM images of ER in PVD soma in Ex[*ser-2*P3::*atln-1*^F161S^] (**g**) and Ex [*ser-2*P3::*atln-1*^I326S^] (**h**) transgenic adult worms. Insets show 2X magnified views. Scale bar, 1 μm. **j** Quantification of 3-way junctions in a 3 μm^2^ region at anterior primary dendrite initiation segment (the dashed boxes in **h** and **i**). Values are mean and error bars are SEM, *n* > 30. ns, not significant; ***p <0.001 (Newman-Keuls multiple comparison test)

Hereditary Spastic Paraplagia (HSP) is a class of diseases characterized by the neuronal degeneration of peripheral nervous system. A number of missense mutations of Atlastin-1 have been reported in HSP patients^46^. The pathogenic mechanisms of these mutations are not fully understood. Interestingly, other HSP genes such as Reticulon and REEP affect ER morphology in *Drosophila* axons^47^. Next we asked if HSP disease mutations of Atlastin-1 affected dendritic ER pattern in PVD. We chose three Atlastin-1 mutations found in HSP patients, F151S, I315S, and S519N, which localized to different domains of Atlastin-1. The corresponding conserved mutation sites in *C. elegans* were F161S, I326S, and S530N, respectively (Fig. 8a). We overexpressed these three disease alleles, the wild-type *atln-1*, and the GTPase defective K80A allele in wild-type worm’s PVD and examined the ER morphology in each genotype. As a control, overexpression of the wild-type *atln-1* did not show any ER phenotype (Fig. 8b, g). However, overexpression of all three HSP alleles and K80A caused reduced ER in the secondary and tertiary branches similar to the phenotypes found in the loss-of-function *atln-1(wy50080)* mutants (Fig. 8c–g). In addition, expression of F161S and I326S also caused striking ER morphology phenotypes in the PVD soma characterized by a reduction of ER networks and increased parallel ER tubules (Fig. 8h–j). Together with the human genetics literatures, these results support the notion that neuronal ER morphology defects contribute to the pathogenesis of HSP.

### ER shaping and HSP genes are required in ER invasion

There are three Atlastin proteins ATL1, ATL2, and ATL3 in mammals, which function redundantly to modulate ER morphology^48^. *C. elegans* genome encodes two Atlastin homologs *atln-1* and *atln-2*. In PVD, *atln-2* was not required for ER morphology as *atln-2(wy50125)* mutant did not show any ER phenotype (Supplementary Fig. 5A and C) and *atln-1(wy50080)*; *atln-2(wy50125)* double mutants did not enhance *atln-1(wy50080)* single mutant’s phenotype (Supplementary Fig. 5B and C). These results suggested that *atln-1* but not *atln-2* was essential in regulating ER morphology in PVD. To address whether *atln-2* is expressed in PVD, we constructed two 2 kb promoters driven GFP (*atln-2*Prom1::GFP and *atln-2*Prom2::GFP) to monitor the expression pattern of different *atln-2* isoforms. Both of the constructs showed expression in a single head neuron but not in PVD (Supplementary Fig. 5D–G). Therefore, the lack of expression in PVD might explain why PVD ER morphology is normal in the *atln-2* mutants.

Aside from ATLs, ER tubules are shaped by the curvature-stabilizing proteins such as Reticulons and REEPs, which are HSP associated proteins^5^. These ER shaping proteins are known to have similar activity and redundant functions^5^. We systematically examined the ER phenotype in mutant alleles for these genes including *yop-1(tm3667)* (REEP5 and REEP6 ortholog), *ret-1(tm390)* (reticulon ortholog), and *T19C3.4(wy50209)* (REEP1-4 ortholog) in PVD. Each mutant showed subtle ER extension defects (Supplementary Fig. 6A, C, D and K). In addition, *lnp-1(wy50227)*, the mutant of another ER shaping protein Lunapark, which functions to stabilize nascent three-way junctions^49^, also showed decreased ER of PVD dendrites (Supplementary Fig. 6E and K). Furthermore, *spas-1(tm683)*, the mutant of another HSP causative gene Spastin^50^ also showed ER morphology defect (Supplementary Fig. 6B and K), although not as severe as the *atln-1*(*wy50080*) mutant.

To explore the relationship between *atln-1* and these genes, we constructed the double mutants between *atln-1* and these genes. Only *ret-1(tm390)*; *atln-1(wy50080)* double mutants enhanced the ER defects of *atln-1* single mutants (Supplementary Fig. 6F–J and L), suggesting *ret-1* and *atln-1* indeed play partially redundant functions to regulate ER extension in PVD dendrites. *yop-1*, *lnp-1*, *spas-1*, and *T19C3.4* did not enhance the phenotype of *atln-1*, which might be due to additional redundant functions of other ER shaping proteins. Together, it suggests that multiple ER shaping and HSP related protein cooperate to regulate ER morphology in neurons.

## Discussion

In this study, we use the *C. elegans* sensory neuron PVD to understand how cell type specific ER morphology is established in dendrites and whether the specific morphology is related to its function in neurons. Our current study adds to the understanding of Atl1 in the following aspects. First; we systemically examine the ER morphology and dynamics in dendrites in vivo. Second; our results suggest that ER and MTs mutually influence each other in dendrites: while MT dynamics and motor proteins mobilize ER extension into dendritic branches, ER also functions to stabilize MTs. Third; we show that complex ER networks at dendritic branch points modulate mitochondria fission and localization. Fourth, we show that ATLN-1 regulates protein homeostasis when UPR is impaired. Fifth, we have examined the impact of many HSP mutant alleles in dendritic ER morphology in our system and show that ER impairment in neurites might be the cause of HSP.

In the PVD soma, ER forms network pattern, similar to non-neuronal cells, while PVD dendrites mainly contain ER tubules. This type of distribution pattern is similar to what has been observed in the mammalian neuron’s cell body and axon^2,15^. Interestingly, we notice that the ER complexity increase with the appearance of small ER networks at dendrite branch points. It is shown that more complex ER networks form at the branch sites of dendrites in culture mammalian neuronal cells^18^. These similarities argue strongly that the ER networks at dendrite branching points are conserved features of dendrites and are likely to have functional significance. In order to understand the structure function relationship between the morphology and function of ER, we search for molecular manipulations that can change critical aspects of ER morphology in neurons. Interestingly, the unbiased genetic approach leads us to the usual suspect of ATLN-1. *atln-1* mutants reduce the complexity of ER likely by preventing tubules fusion and three-way junctions formation. In *atln-1* mutants, ER has less branched network structures and more parallel tubules. This change is apparent at both cell body and the dendritic branch points. Next, we ask if these ER morphology changes lead to functional deficits in *atln-1* mutants.

First, related to the reduction of ER networks at dendrite branch points, we find reduced ER tubules in the corresponding dendritic branches. Interestingly, in *atln-1* mutants, ER tubules’ growth into dendritic branches is not affected. Instead, the retraction of tubules from dendrite branches is increased compared with wild-type animals. These results suggest that the ER networks might be required for stabilizing the ER tubules that extend into the 2° dendrites. It is conceivable that the ER networks at the branch sites modulate the ER membrane tension, which affects the stability of ER tubules.

Second, we find that mitochondria are enriched at the dendrite branch points. More than half of the mitochondria are localized nearly to branch points, which is dramatically different from a random pattern. This branch point enrichment is reduced in *atln-1* mutants, suggesting that the ER networks might contribute to anchoring of mitochondria either through physical hindrance of trafficking or specific molecular interactions between mitochondria and ER. In wild-type animals, we also observe that mitochondria undergo fission preferentially at the branch points. Since ER-mitochondria contact has been shown to facilitate mitochondria fission^41^, it is likely that the ER networks at the branch points provide a platform for mitochondria anchoring and fission. Together with the previous evidences showing the complex ER structure at branch points enriches protein synthesis and secretion machinery^18^, our data broadens the potential function of branch point ER networks.

Third, related to the somatic ER networks, we find that the level of the dendrite receptor protein DMA-1 is significantly reduced in the *atln-1* mutant under ER stress conditions. Consequently, the dendrite branching phenotype of *ire-1* mutant is significantly enhanced by the *atln-1* mutation. Since the vast majority of ribosomes are found in the soma (Supplementary Fig. 7), we postulate this defect of *atln-1* mutants is due to the ER networks defect in the soma. Together, these evidences suggest that the ER networks morphology in the neuronal soma and at dendrite branch points are important for several aspects of neuronal cell biology.

In wild-type animals, ER tubules extend from the branch-site networks into the 2° branches using MTs dependent mechanisms. The extension of the ER requires the presence of MTs within the branches and is likely dependent on MT motor proteins^34^. The distribution of ER tubules in dendritic branches highly correlates with the MTs distribution, suggesting that the growth of ER and MTs are related. In fibroblasts, the interaction between ER and MTs is an important determinant of ER morphology^54^. When ER is bound to MTs in fibroblasts, CLIMP63 promotes extension of ER tubules to the cell periphery along MT tracks^51,52^. However, we find that both ER and MTs extension into the branches are defective in *atln-1* mutant. And our data show that ATLN-1 likely directly affects ER morphology and indirectly affects MT dynamics. For the ER extension defect of *atln-1* mutant, Our results show that this phenotype correlates with the reduced complexity of ER networks at the branching points (Supplementary Fig. 3), which may directly depend on the function of ATLN-1. For the MT extension defect in *atln-1* mutants, we speculate it’s a secondary effect of defective ER since we find that ER and MTs could mutually affect each other in our system.

MTs are required for the entry of ER into the PVD dendritic branches. This is consistent with many previous studies in the field. Our unpublished data also show that: (1) ER entry into the 2° and 3° branches is strictly dependent on the presence of MTs in the same branches. (2) ER could enter the PVD 2° dendrites along static MTs, likely driven by MTs based motors. (3) ER could also enter together with the growing tip of MTs. Both motor mediated and tip attachment mechanisms are consistent with the mechanisms of ER movement in non-neuronal cells. (4) ER could also retract from branches, which could happen in the absence of MTs. Therefore, ER needs MTs to grow but ER retraction is not dependent on MTs.

Since some branches contain MTs and others do not, the PVD dendrites provide us with an experimental system to ask whether ER can also affect the dynamic behavior of MTs. In wild-type animals, when we compare MTs in the branches with or without ER, we find that MTs grow less and shrink less in ER positive branches, indicating that the presence of ER makes MTs more stable (less dynamic) (Fig. 5a–e). To further corroborate these results, we examine the PTRN-1 puncta as a proxy to MT numbers in –ER and +ER branches. We find that there are more PTRN-1 puncta in the +ER branches, indicating that there are more MTs in the +ER branches (Fig. 5f, g). The fact that *atln-1* mutants do not change the dynamic behavior of MTs in –ER branches suggests an indirect role of ATLN-1 on MT dynamic regulation. Additionally, *atln-1* mutants show slightly more stable MT plus-ends in the +ER branches compared to the wild-type control. This effect is subtle but is statistically significant and could be rescued by cell autonomous expression of wild-type ATLN-1. Consistent with our data, Atlastin-1 has been shown to physically interact with the N terminal of MT-severing protein Spastin in *Drosophila* muscle, which negatively regulates MT stability^53^. Taken together, these data indicate that ER might be able to stabilize MTs in the dendrites with an unknown mechanism.

Atlastin-1/SPG3A is a well-known factor in HSP, which is supported by the discoveries of many Atlastin-1 mutations in HSP patients. Introducing several mutant alleles to worms show very similar phenotype in PVD soma and dendrites as *atln-1* loss-of-function mutants, suggesting the conserved function of Atlastin-1 in controlling dendrite and neuronal somatic ER morphology might contribute to HSP pathogenesis. Furthermore, mutants of several other HSP genes including YOP-1 (REEP), RET-1(reticulon) also show ER morphology defects in dendrite, indicating that neuronal ER morphogenesis defect might be a common pathological feature of HSP.

## Methods

### Strains and genetics

The wild-type strain N2 Bristol and all the mutants were cultured on the nematode growth medium (NGM) plates seeded with *Escherichia coli* OP50 at 20 °C following the standard protocol (Brenner, 1974). Standard cloning procedure of Clontech In-Fusion PCR Cloning System was used to construct all the plasmids. PCR products were produced by Phusion DNA polymerase (New England Biolabs) or TransStart FastPfu DNA Polymerase (Transgen Biotech) or High-Fidelity Master Mix (TsingKe Biotech) following standard procedures. All the strains and plasmids used are listed in Supplementary Table 1, Supplementary Table 2, Supplementary Table 3, and Supplementary Table 4.

### Fluorescent imaging, time-lapse imaging, Kymograph analysis

For fluorescent imaging, worms were immobilized with 1 mg/ml levamisole in M9 buffer and transferred to a small glass slide or dish, then covered by a 3% (w/v) agar pad. Images were acquired using a 40X objective on a Zeiss Axio Observer Z1 microscope equipped with a 10X, 63X and 100X objective, an electron-multiplying charge-coupled device camera (Andor), a spinning-disk confocal scan head (Yokogawa CSU-X1 Spinning Disk Unit), and the 488- and 561 nm lines of a Sapphire CW CDRH USB Laser System. For ER dynamic analysis in Fig. 3, 200 frames were taken for each movie at the speed of 1.54 frames per second. For MT dynamic analysis in Fig. 5, 150 frames were taken for each movie at the speed of 1.19 frames per second. Movies were taken from PVD tertiary dendrites. For mitochondria fission analysis in Fig. 6, 120 frames were taken for each movie at the speed of 0.2 frames per second. Movies were taken from the view in which PVD primary dendrite contained apparent long tubular mitochondria.

To measure the frequency of MT dynamic events, the term “catastrophe” is defined as the transition between polymerization and de-polymerization, whereas “rescue” describes the reverse transition. The frequency of dynamic events was calculated by counting the number of dynamic events dividing recording time. The growth and retraction length of ER was measured by the horizontal distance between adjacent transitions in kymographs. Movies and kymographs were generated and analyzed by ImageJ.

### Quantification and statistics

For ER, dendritic and MT 2° branches quantification, worms at distinct stage were checked under 40X or 63X objectives using an Axio Imager M2 microscope (Carl Zeiss). For the quantification of 3-way junctions in PVD soma in Figs. 2 and 8, a most recognizable region of 3 μm^2^ (63 pixel × 31 pixel) at anterior primary dendrite initiation segment was selected, and this region would cover as more ER area as possible. Statistics analysis was performed using student’s t test, Tukey’s multiple comparison test, Newman-Keuls multiple comparison test or Chi-square test. Statistical analyses were generated and performed using Microsoft Excel and Prism 6 (GraphPad Software).

### 3D-SIM super-resolution microscopy and image analysis

For 3D-SIM images, worms were immobilized with 3 mg/ml levamisole in M9 buffer and transferred to a small glass slide or dish, then covered by a 4–5% (w/v) agar pad. A half hour later, images were acquired on the DeltaVision OMX V3 imaging system (GE Healthcare) with a 100X/1.4NA oil objective (Olympus UPlanSApo), solid-state multimode lasers (488, 405, 561 nm) and electron-multiplying CCD (charge-coupled device) camera (Evolve 512 × 512, Photometrics). Serial Z-stack sectioning was done at 125 nm intervals for SIM mode. To obtain optimal images, immersion oils with refractive indices of 1.520 were used for PVD cell body or branch point on glass coverslips. The microscope is routinely calibrated with 100 nm fluorescent spheres to calculate both the lateral and axial limits of image resolution. SIM image stacks were reconstructed using softWoRx 6.1.1 (GE Healthcare) with the following settings: pixel 39.5 nm; channel-specific optical transfer function; Wiener filter constant 0.0010; discard Negative Intensities background; drift correction with respect to first angle; custom K0 guess angles for camera positions. The reconstructed images were further processed for maximum-intensity projections with softWoRx 6.1.1. Pixel registration was corrected to be less than 1 pixel for all channels using 100 nm Tetraspeck beads.

### GTPase activity assay

Full-length, codon-optimized wild-type and mutant Drosophila ATL were expressed in in BL21 (DE3) cells as GST fusions. Expression was induced by the addition of 0.1 mM IPTG and continued for 24 h at 16 °C. Cells were lysed in A100 buffer [25 mM Hepes (pH 7.5), 100 mM KCl, 1 mM EDTA, 2 mM β-mercaptoethanol, and 10% (vol/vol) glycerol]. The membranes were pelleted by centrifugation and solubilized in Triton X-100. The GST fusion proteins were isolated with glutathione sepharose beads (GE Healthcare), washed, and eluted with 10 mM glutathione in A100 buffer with 0.1% Triton X-100. The GST tag was cleaved by Prescission protease (GE Healthcare) and was removed with glutathione agarose. GTPase assays were done with the Enzchek phosphate assay kit (Invitrogen). Reactions were performed in a 200 μL volume with 10 μL of 20× reaction buffer (1 M Tris·HCl/20 mM MgCl2, pH 7.5/2 mM sodium azide), 200 mM 2-amino-6-mercapto-7-methylpurine riboside, 1 unit/mL purine nucleoside phosphorylase (PNP), 2 μM ATL protein, or the indicated mutants, and incubated for 10 min at 37 °C in a 96-well plate (Nunc). Reactions were initiated by the addition of 0.5 mM GTP. The absorbance at 360 nm was measured every 31 s over 40 min at 37 °C by using a microplate reader (infinite M200 PRO). Rates of phosphate release were then calculated based on a standard curve.

### Liposomes tethering and lipids mixing assay

Detergent-mediated reconstitution was used to integrate dmATL into preformed donor liposomes [82:15:1.5:1.5 mole percent of 1-palmitoyl-2-oleoyl-sn-glycero-3-phosphocholine (POPC):1, 2-dioleoyl-sn-glycero-3-phosphoserine (DOPS): NBD-1, 2-dipalmitoyl-sn-glycero-3-phosphoethanolamine (DPPE): rhodamine-DPPE] and acceptor liposomes (85:15 mole percent of POPC:DOPS). Lipids in chloroform were dried under a stream of N2 gas followed by further drying in a vacuum for 30 min. The lipid films were then re-suspended in A100 buffer to a final total lipid concentration of about 10 mM. Large unilamellar vesicles (LUVs) were formed by ten freeze-thaw cycles in liquid N2 and room temperature water. Uniform-sized LUVs were formed by extrusion through polycarbonate filters with 100-nm pore size (Avanti Polar Lipids). Wild-type and mutant Drosophila ATL proteins were mixed with preformed liposomes at an effective detergent to lipid ratio of ~1:1. Proteins and lipids were allowed to mix for 1 h at 4 °C. Detergent was removed by adding BioBeads SM-2 adsorbant beads (BioRad) five times every hour, and incubating overnight after the final addition of beads. Insoluble protein aggregates were pelleted by centrifugation of the samples in an Eppendorf microcentrifuge. For Liposomes tethering assay, GTP was added at 1 mM concentration to proteoliposomes containing WT and mutant ATL. Absorbance at 405 nm was measured on an infinite M200 PRO Microplate Reader. The absorbance before nucleotide addition was set to zero. For lipids mixing assay, donor and acceptor vesicles were mixed at a 1:3 ratio in A100 supplemented with 5 mM MgCl_2_. GTP was added to 1 mM to start the fusion reaction, and the fluorescence intensity of NBD was monitored with an excitation of 460 nm and emission of 538 nm using a SpectraMax M5 Microplate Reader or Biotek Synergy 4 plate reader. The maximal NBD fluorescence was determined after addition of dodecyl maltoside. The initial NBD fluorescence prior to nucleotide addition was set to zero, and the initial background fluorescence was subtracted from the raw fluorescence readings, and these values are expressed as percentages of the maximum fluorescence after detergent addition.

### Reporting summary

Further information on experimental design is available in the Nature Research Reporting Summary linked to this article.

## Supplementary information

Supplementary Information

Reporting Summary

## Data Availability

All source data and all results Figures in the Main and Supplementary Information sections of the Article are available directly from the authors. Extended results are available as a Supplementary Information file.

## References

[1] Baumann O, Walz B (2001). Endoplasmic reticulum of animal cells and its organization into structural and functional domains. Int. Rev. Cytol..

[2] Shibata Y, Voeltz GK, Rapoport TA (2006). Rough sheets and smooth tubules. Cell.

[3] Goyal U, Blackstone C (2013). Untangling the web: mechanisms underlying ER network formation. Biochim. Biophys. Acta.

[4] Hu J, Prinz WA, Rapoport TA (2011). Weaving the web of ER tubules. Cell.

[5] Zhang H, Hu J (2016). Shaping the Endoplasmic Reticulum into a Social Network. Trends Cell Biol..

[6] Voeltz GK, Prinz WA, Shibata Y, Rist JM, Rapoport TA (2006). A class of membrane proteins shaping the tubular endoplasmic reticulum. Cell.

[7] Hu J (2009). A class of dynamin-like GTPases involved in the generation of the tubular ER network. Cell.

[8] Orso G (2009). Homotypic fusion of ER membranes requires the dynamin-like GTPase atlastin. Nature.

[9] Zhang M, Hu J (2013). Homotypic fusion of endoplasmic reticulum membranes in plant cells. Front. Plant Sci..

[10] Shibata Y (2010). Mechanisms determining the morphology of the peripheral ER. Cell.

[11] Chen S, Novick P, Ferro-Novick S (2012). ER network formation requires a balance of the dynamin-like GTPase Sey1p and the Lunapark family member Lnp1p. Nat. Cell Biol..

[12] Chang J, Lee S, Blackstone C (2013). Protrudin binds atlastins and endoplasmic reticulum-shaping proteins and regulates network formation. Proc. Natl Acad. Sci. USA.

[13] Bennett PM (2012). From myofibril to membrane; the transitional junction at the intercalated disc. Front Biosci. (Landmark Ed.).

[14] West M, Zurek N, Hoenger A, Voeltz GK (2011). A 3D analysis of yeast ER structure reveals how ER domains are organized by membrane curvature. J. Cell Biol..

[15] Yalcin B (2017). Modeling of axonal endoplasmic reticulum network by spastic paraplegia proteins. eLife.

[16] Cooney JR, Hurlburt JL, Selig DK, Harris KM, Fiala JC (2002). Endosomal compartments serve multiple hippocampal dendritic spines from a widespread rather than a local store of recycling membrane. J. Neurosci..

[17] Spacek J, Harris KM (1997). Three-dimensional organization of smooth endoplasmic reticulum in hippocampal CA1 dendrites and dendritic spines of the immature and mature rat. J. Neurosci..

[18] Cui-Wang T (2012). Local zones of endoplasmic reticulum complexity confine cargo in neuronal dendrites. Cell.

[19] Schule R, Schols L (2011). Genetics of hereditary spastic paraplegias. Semin. Neurol..

[20] Noreau A, Dion PA, Rouleau GA (2014). Molecular aspects of hereditary spastic paraplegia. Exp. Cell Res..

[21] Guelly C (2011). Targeted high-throughput sequencing identifies mutations in atlastin-1 as a cause of hereditary sensory neuropathy type I. Am. J. Hum. Genet..

[22] De Gregorio C, Delgado R, Ibacache A, Sierralta J, Couve A (2017). Drosophila Atlastin in motor neurons is required for locomotion and presynaptic function. J. Cell Sci..

[23] Summerville JB (2016). The effects of ER morphology on synaptic structure and function in Drosophila melanogaster. J. Cell Sci..

[24] Shih YT, Hsueh YP (2016). VCP and ATL1 regulate endoplasmic reticulum and protein synthesis for dendritic spine formation. Nat. Commun..

[25] Halevi S (2002). The C. elegans ric-3 gene is required for maturation of nicotinic acetylcholine receptors. EMBO J..

[26] Smith CJ (2010). Time-lapse imaging and cell-specific expression profiling reveal dynamic branching and molecular determinants of a multi-dendritic nociceptor in C. elegans. Dev. Biol..

[27] Rolls MM, Hall DH, Victor M, Stelzer EH, Rapoport TA (2002). Targeting of rough endoplasmic reticulum membrane proteins and ribosomes in invertebrate neurons. Mol. Biol. Cell.

[28] Liu OW, Shen K (2011). The transmembrane LRR protein DMA-1 promotes dendrite branching and growth in C. elegans. Nat. Neurosci..

[29] Dong X, Liu OW, Howell AS, Shen K (2013). An extracellular adhesion molecule complex patterns dendritic branching and morphogenesis. Cell.

[30] Tsalik EL (2003). LIM homeobox gene-dependent expression of biogenic amine receptors in restricted regions of the C. elegans nervous system. Dev. Biol..

[31] Bian X (2011). Structures of the atlastin GTPase provide insight into homotypic fusion of endoplasmic reticulum membranes. Proc. Natl Acad. Sci. USA.

[32] Faust JE (2015). The Atlastin C-terminal tail is an amphipathic helix that perturbs the bilayer structure during endoplasmic reticulum homotypic fusion. J. Biol. Chem..

[33] Li X (2017). Large scale three-dimensional reconstruction of an entire Caenorhabditis elegans larva using AutoCUTS-SEM. J. Struct. Biol..

[34] Waterman-Storer CM, Salmon ED (1998). Endoplasmic reticulum membrane tubules are distributed by microtubules in living cells using three distinct mechanisms. Curr. Biol..

[35] Maniar TA (2011). UNC-33 (CRMP) and ankyrin organize microtubules and localize kinesin to polarize axon-dendrite sorting. Nat. Neurosci..

[36] Lacroix B (2014). In situ imaging in C. elegans reveals developmental regulation of microtubule dynamics. Dev. Cell..

[37] Yogev S, Cooper R, Fetter R, Horowitz M, Shen K (2016). Microtubule organization determines axonal transport dynamics. Neuron.

[38] Richardson CE (2014). PTRN-1, a microtubule minus end-binding CAMSAP homolog, promotes microtubule function in Caenorhabditis elegans neurons. eLife.

[39] Zou W (2016). A multi-protein receptor-ligand complex underlies combinatorial dendrite guidance choices in C. elegans. eLife.

[40] Park SH, Zhu PP, Parker RL, Blackstone C (2010). Hereditary spastic paraplegia proteins REEP1, spastin, and atlastin-1 coordinate microtubule interactions with the tubular ER network. J. Clin. Invest..

[41] Korobova F, Ramabhadran V, Higgs HN (2013). An actin-dependent step in mitochondrial fission mediated by the ER-associated formin INF2. Science.

[42] Hirabayashi Y (2017). ER-mitochondria tethering by PDZD8 regulates Ca(2+) dynamics in mammalian neurons. Science.

[43] Friedman JR (2011). ER tubules mark sites of mitochondrial division. Science.

[44] Chen Y, Brandizzi F (2013). IRE1: ER stress sensor and cell fate executor. Trends Cell Biol..

[45] Wei X (2015). The unfolded protein response is required for dendrite morphogenesis. eLife.

[46] Ulengin I, Park JJ, Lee TH (2015). ER network formation and membrane fusion by atlastin1/SPG3A disease variants. Mol. Biol. Cell.

[47] O’Sullivan NC, Jahn TR, Reid E, O’Kane CJ (2012). Reticulon-like-1, the Drosophila orthologue of the hereditary spastic paraplegia gene reticulon 2, is required for organization of endoplasmic reticulum and of distal motor axons. Hum. Mol. Genet..

[48] Hu X, Wu F, Sun S, Yu W, Hu J (2015). Human atlastin GTPases mediate differentiated fusion of endoplasmic reticulum membranes. Protein Cell.

[49] Chen S (2015). Lunapark stabilizes nascent three-way junctions in the endoplasmic reticulum. Proc. Natl Acad. Sci. USA.

[50] Hazan J (1999). Spastin, a new AAA protein, is altered in the most frequent form of autosomal dominant spastic paraplegia. Nat. Genet..

[51] Klopfenstein DR, Kappeler F, Hauri HP (1998). A novel direct interaction of endoplasmic reticulum with microtubules. EMBO J..

[52] Vedrenne C, Klopfenstein DR, Hauri HP (2005). Phosphorylation controls CLIMP-63-mediated anchoring of the endoplasmic reticulum to microtubules. Mol. Biol. Cell.

[53] Lee M (2009). Drosophila Atlastin regulates the stability of muscle microtubules and is required for synapse development. Dev. Biol..

[54] Vedrenne C, Hauri HP (2006). Morphogenesis of the endoplasmic reticulum: beyond active membrane expansion. Traffic.

