# Supplementary material for: Atlastin-1 regulates morphology and function of endoplasmic reticulum in dendrites
Source: Nat Commun. 2019 Feb 4;10:568. doi: 10.1038/s41467-019-08478-6 (PMC6362286; doi:10.1038/s41467-019-08478-6)
Supplement: Supplementary file 1 — Supplementary Information [file 41467_2019_8478_MOESM1_ESM.pdf]

Liu et al. "Atlastin-1 Regulates Morphology and Function of Endoplasmic Reticulum in Dendrites"

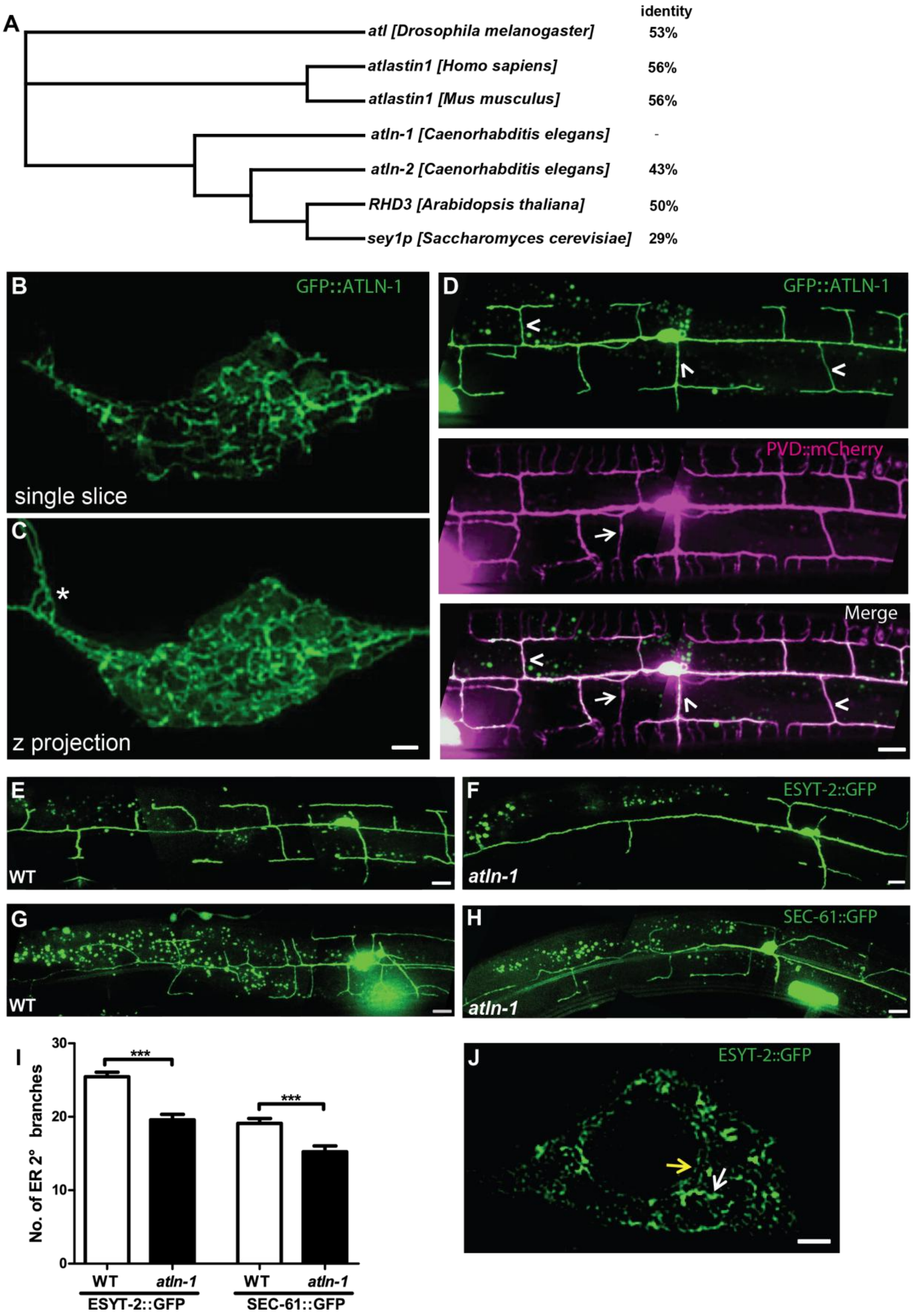

Supplementary Figure 1. ATLN-1 functions in ER invasion into PVD dendrites. (A) Phylogenetic tree of Atlastin proteins (created by BioEdit, ClustalW and TreeView software) and protein sequence identity. **(B-C)** Representative single focal plane (B) and maximum-intensity -projection (C) 3D-SIM image of subcellular GFP::ATLN-1 in PVD soma. Asterisk marks a complex ER network at a PVD dendrite branch point. Scale bar, 1μm. **(D)** Subcellular distribution of GFP::ATLN-1 in PVD dendrites. Arrows show PVD 2° branches without ER and arrowheads indicate 2° branches with ER. Scale bar, 10μm. **(E-H)** Representative confocal images of ER

morphology, visualizing by ESYT-2::GFP (E-F) and SEC-61::GFP (G-H) as ER markers in PVD, in WT (E, G) and *atln-1* mutant (F, H). Adult worms are shown. Scale bars, 10µm. **(I)** Quantification of the number of ER in 2 °branches. Values are mean and error bars are SEM, n>30. ***p<0.001 (two-sided student's t test). **(J)** A representative 3D-SIM image of subcellular ESYT-2::GFP in PVD soma (single focal plane). Yellow arrow indicates the nuclear membrane and white arrow indicates a tubular network. Scale bar, 1µm.

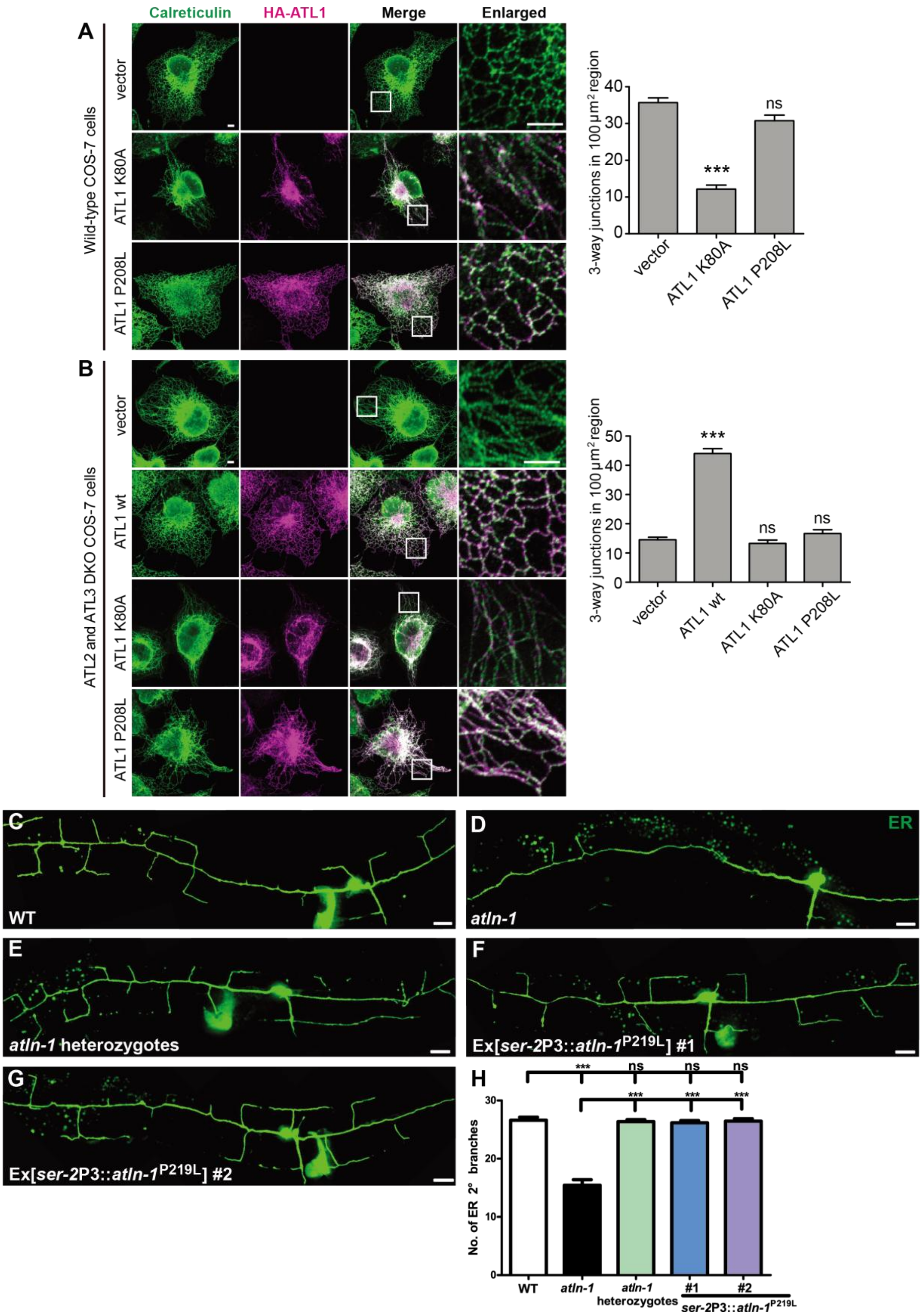

Supplementary Figure 2. *atln-1(wy50080)* is a loss-of-function allele. (A) Wild-type COS-7

cells were transfected with vector, HA-ATL1 (K80A) or HA-ATL1 (P208L). 24h after transfection, cells were fixed and stained with anti-calreticulin and anti-HA antibodies. Representative confocal images are shown. The right panel is the quantification of 3-way junctions in a 100 μm^2 region away from the nucleus. Scale bars, 5μm. Values are mean and error bars are SEM, at least 20 different cells per group were examined. ns, not significant; ***p<0.001 (Newman-Keuls multiple comparison test). **(B)** ATL2 and ATL3 DKO COS-7 cells were transfected with vector, HA-ATL1 (WT), HA-ATL1 (K80A) or HA-ATL1 (P208L). After 24h, cells were fixed and stained with anti-calreticulin and anti-HA antibodies. Representative confocal images are shown. The right panel is the quantification of 3-way junctions in a 100 μm^2 region away from the nucleus. Scale bars, 5μm. Values are mean and error bars are SEM, at least 20 different cells per group were examined. ns, not significant; ***p<0.001 (Newman-Keuls multiple comparison test). **(C-G)** Representative confocal images of ER morphology in PVD in WT (C), *atln-1 -/-* (D), *atln-1* +/- (E), Ex[*ser-2*P3::*atln-1*^{P219L}]#1 (F), and Ex[*ser-2*P3::*atln-1*^{P219L}] #2 (G) worms at adult stage. Scale bars, 10μm. **(H)** Quantification of the number of ER in 2° branches. Values are mean and error bars are SEM, n=46 for each genotype. ns, not significant; ***p<0.001 (Tukey's multiple comparison test).

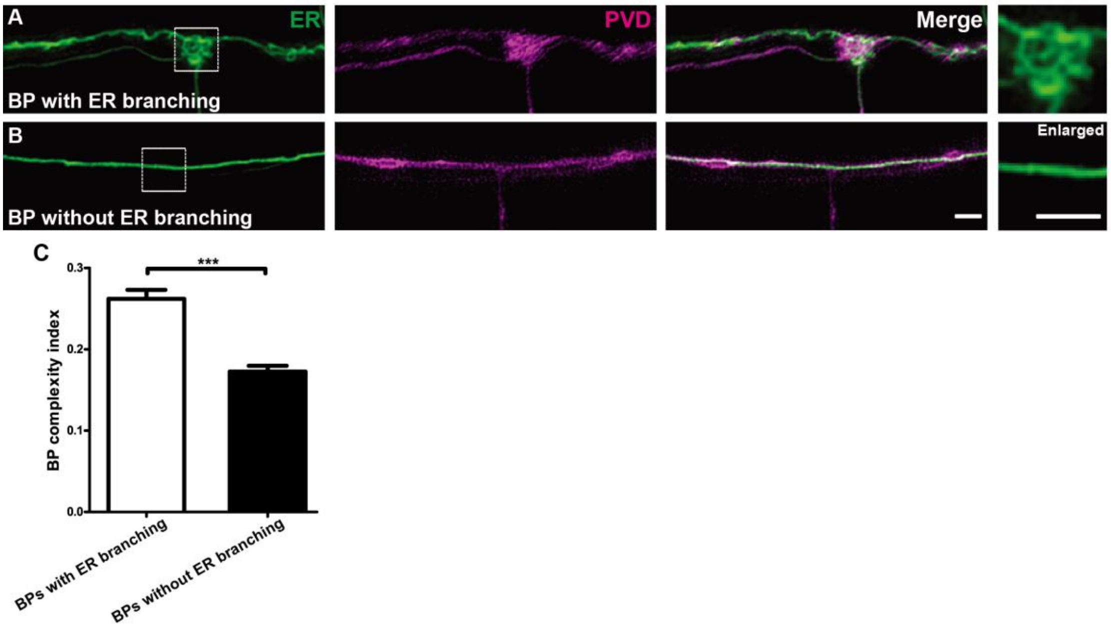

Supplementary Figure 3. Complex ER networks at dendritic BPs stabilize ER tubules. (A-B) Representative 3D-SIM images of ER (GFP::SP12) complex structure at BPs with (A) or without ER tubules (B) in 2° branches. Scale bars, 1μm. (C) Quantification of ER complexity index of BPs with or without ER tubules in 2° branches. Values are mean and error bars are SEM, at least 70 BPs from more than 30 worms are quantified. ***p<0.001 (two-sided student's t test).

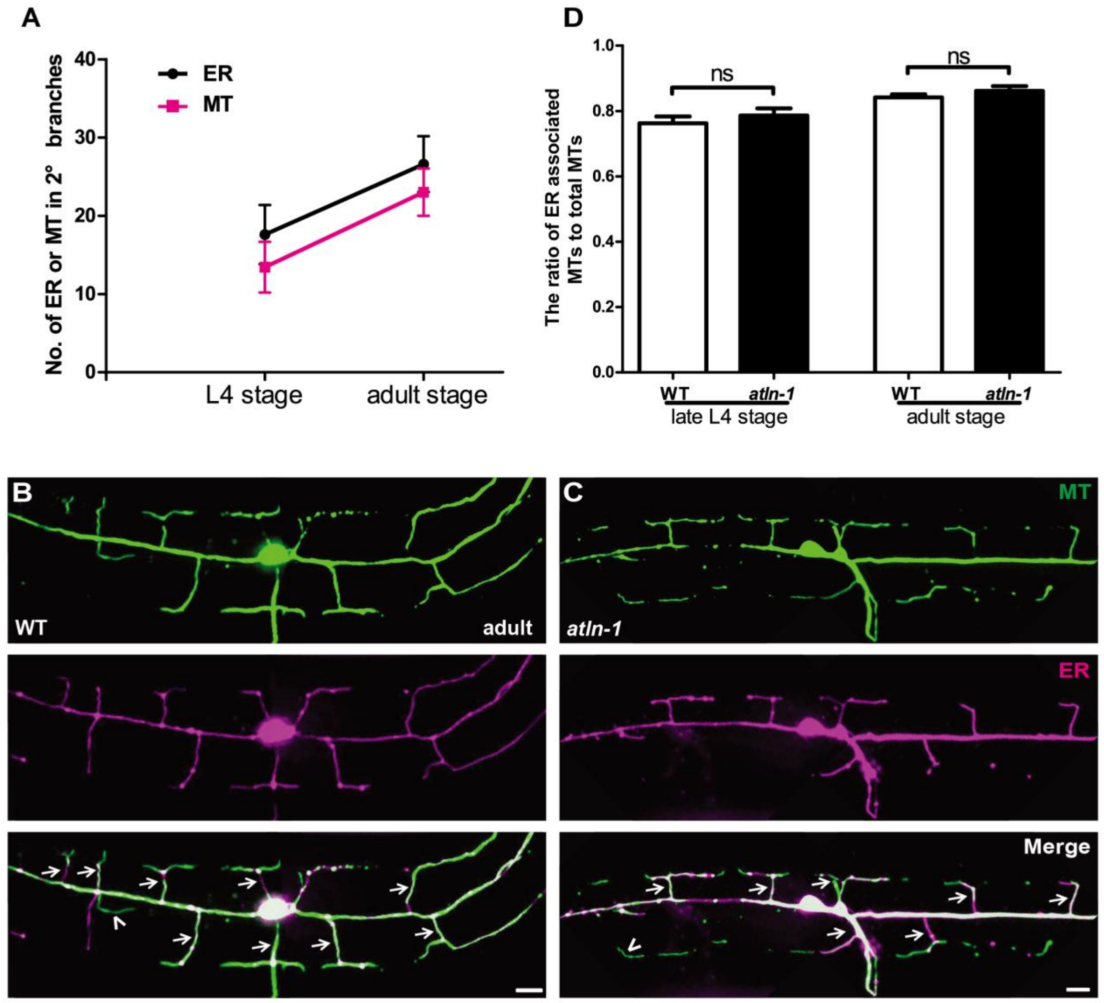

Supplementary Figure 4. MTs and ER colocalize in dendritic branches. (A) A line chart showing synchronized increase of ER and MT in dendritic branches. Values are mean and error bars are SD, n>40 for each genotype at different developmental stages. **(B-C)** Representative confocal images of MT (TBA-1::GFP) and ER (mCherry::SP12) morphology of PVD neuron in WT (B) and *atln-1* (C) worms at adult stage. Arrows point to MTs in 2 ° branches which are colocalized with ER and arrowheads point to MTs in 2 °branches which are not colocalized with ER. Scale bars, 10μm. **(D)** Quantification of the percentage of MTs which are colocalized with ER in 2 ° branches. Values are mean and error bars are SEM, n>30. ns, not significant (two-sided student's t test).

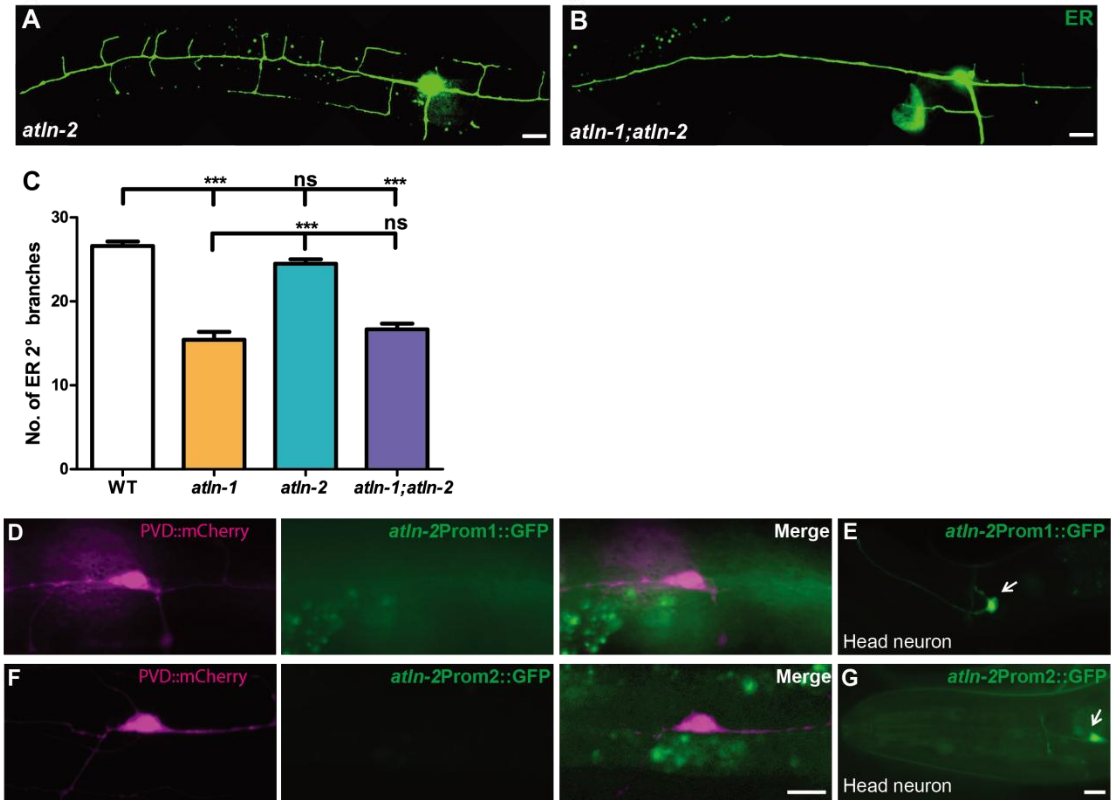

Supplementary Figure 5. *atln-1* but not *atln-2* affects ER morphogenesis in PVD. (A and B) Representative confocal images of ER (GFP::SP12) morphology in PVD in *atln-2*(*wy50125*) (A) and *atln-1*; *atln-2* double mutants (B) worms at adult stage. Scale bars, 10μm. **(C)** Quantification of the number of ER in 2 ° branches. Values are mean and error bars are SEM, n=46 for each genotype. ns, not significant; ***p<0.001 (Tukey's multiple comparison test). **(D-G)** Images of transgenic worms expression PVD::mCherry together with *atln-2*Prom1::GFP (D) and *atln-2*Prom2:: GFP (F). Head region fluorescence in the same transgenic animals (E and G). Scale bars, 10μm.

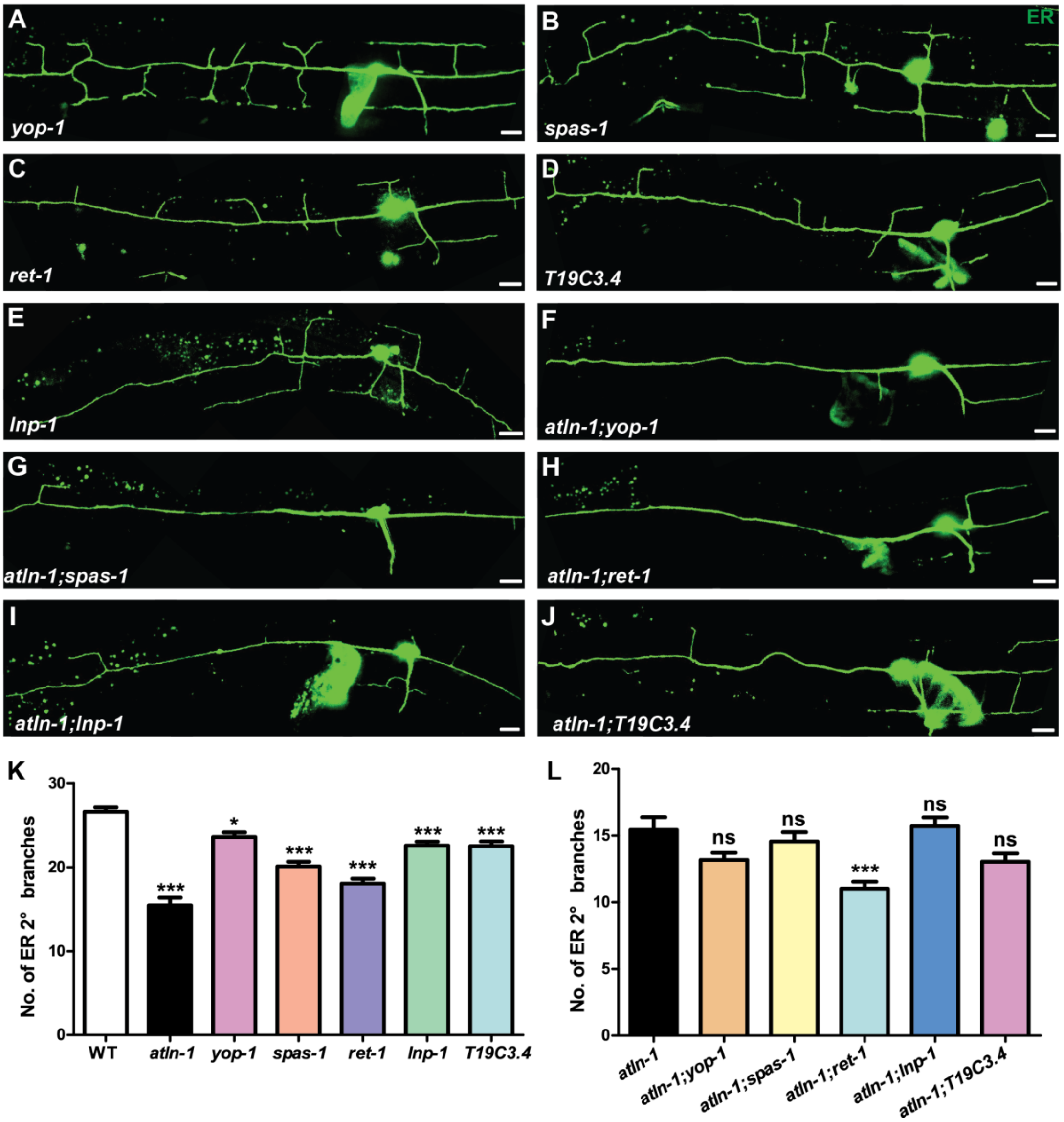

Supplementary Figure 6. Multiple ER shaping and HSP related genes affect ER extension. (A-J) Representative confocal images of ER morphology in PVD in *yop-1*(*tm3667*) (A), *spas-1*(*tm683*) (B), *ret-1*(*tm390*) (C), *T19C3.4*(*wy50209*) (D), *lnp-1*(*wy50227*) (E), *atln-1*; *yop-1* (F), *atln-1*; *spas-1* (G), *atln-1*; *ret-1* (H), *atln-1*; *lnp-1* (I), and *atln-1*; *T19C3.4*(*wy50209*) (J) worms at adult stage. Scale bars, 10μm. **(K-L)** Quantification of ER 2 °branches number. Values are mean and error bars are SEM, n=46 for each genotype. ns, not significant; *p<0.05; ***p<0.001 (Tukey's multiple comparison test).

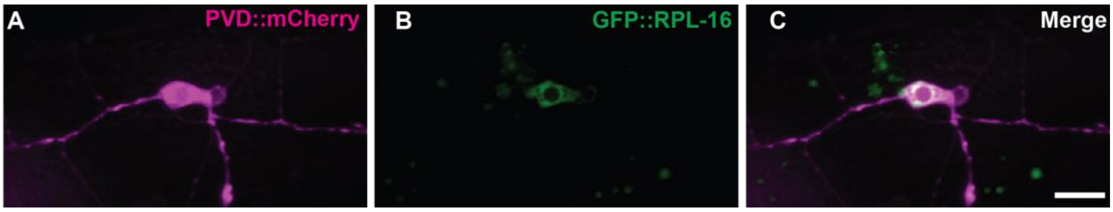

Supplementary Figure 7. Almost all ribosomes are located in PVD soma. (A-C) Ribosomes distribution in PVD (A) at adult stage, visualized by GFP::RPL-16 (large ribosomal subunit protein) as ribosome marker (B), and merged image (C). Scale bar, 10μm.

Supplementary Table 1.Mutant Alleles Used in This Study

Allele	Reference (sources)
atln-1(wy50080)	EMS screen
tba-1(ok1135)	From CGC
ire-1(ok799)	(Xing Wei et al.,1998)
atln-2(wy50125)	cas9 editing
yop-1(tm3667)	From CGC
spas-1(tm683)	From CGC
ret-1(tm390)	From CGC
T19C3.4(wy50209)	cas9 editing
lnp-1(wy50227)	cas9 editing

Supplementary Table 2.Integrated Transgenes Used in This Study

**Allele**	**Chromosome**	**Constructs**	**Co-injection marker**
wyIs500057	X	pXM122(10ng/ul),pOL036(25ng/ul)	P*odr-1*::GFP
wyIs592	III	pOL020(20ng/ul)	P*odr-1*::RFP
wyIs706	X	P*des2*::GFP-gsgs::TBA-1	*ser-2*P3::mCherry
wyIs50070	?	pXM190(10ng/ul),*ser-2*P3::GFP::TBA-1(15ng/ul)	P*odr-1*::RFP
wyIs50054	II	pXM289(20ng/ul),pOL036(5ng/ul)	P*odr-1*::GFP
wyIs740	V	pXD300(10ng/ul),	P*odr-1*::GFP

Supplementary Table 3. Extrachromosomal Arrays Used in This Study

**Allele**	**Constructs**	**Co-injection marker**
wyEx50684	pXM317(10ng/ul)	P*myo-2*::RFP(1ng/ul)
wyEx50685	pXM341(10ng/ul)	P*myo-2*::RFP(1ng/ul)
wyEx50686	pXZ26 (20ng/ul)	P*myo-2*::RFP(2ng/ul)
wyEx50687	pXM341(30ng/ul)	P*myo-2*::RFP(1.5ng/ul)
wyEx50688	pXM401(25ng/ul)	P*odr-1*::RFP(50ng/ul)
wyEx50689	pXM402(25ng/ul)	P*myo-2*::RFP(1.5ng/ul)
wyEx50690	pXM403 (25ng/ul)	P*myo-2*::RFP(1.5ng/ul)
wyEx50257	pXM173(2ng/ul),pOL036(5ng/ul)	P*odr-1*::RFP(50ng/ul)
wyEx50649	pXM477 (5ng/ul),pOL036 (10ng/ul)	P*odr-1*::RFP(50ng/ul)
wyEx50313	pXM199(5ng/ul),pOL036(20ng/ul)	P*odr-1*::RFP(50ng/ul)+<br>P*odr-1*::GFP(50ng/ul)

Supplementary Table 4. Plasmid Used in This Study

Plasmid	Genotype
pXM122	*ser-2*P3(1.6k)::GFP::C34B2.10 (SP12)
pOL036	*ser-2*Prom3::mCherry
pOL020	*ser-2*Prom3::GFP
pXM190	*ser-2*P3(1.6k)::mCherry::C34B2.10
pXM289	ser-2P3(1.6)::TOMM-20(1-54aa)::GFP
pXD300	ser-2Prom3::DMA-1::GFP::DMA-1_cyto
pXM317	*ser-2*P3::ATLN-1
pXM341	*ser-2*P3::ATLN-1K80A
pXZ26	*ser-2*P3::YOP-1
pXM401	*ser-2*P3::ATLN-1F161S
pXM402	*ser-2*P3::ATLN-1FI326S
pXM403	*ser-2*P3::ATLN-1S530N
pXM173	*ser-2*P3::GFP::ATLN-1
pXM199	*ser-2*P3::ESYT-2::GFP
pXM477	*ser-2*P3::SEC-61::GFP

Supplementary Methods

Cloning and plasmids

The plasmid encoding HA-ATL1 was generated by cloning full-length human ATL1 amplified from Myc-ATL1[1] into pcDNA4/TO (Invitrogen) and an HA tag was inserted by PCR. The construct encoding HA-ATL1 K80A and HA-ATL1 P208L were generated using the QuikChange Site-Directed Mutagenesis Kit (Stratagene).

Cell culture

COS-7 cells were obtained from ATCC (CRL-1651). Cells were cultured in DMEM containing 10% fetal bovine serum (10099-141, Gibco), at 37℃ and 5% CO_2. ATL2 and ATL3 DKO cells were generated using a CRISPR/Cas9 genome-editing strategy[2]. sgRNAs (ATL2: 5'-GACGAGATCTTAACATAGTAGTGG-3', ATL3: 5'-GTTTT CACTGTGGAGAAGCCAGG-3') containing oligonucleotides were introduced into the pX330 vector. CRISPR/Cas9 plasmids were transiently transfected into COS7 cells along with pLKO.1-puro at 1:1:1 ratio using TurboFect transfection reagent (Thermo). 24 hours after transfection, cells were treated with 1 μg/ml puromycin to select for cells expressing the sgRNAs. The cells were then sorted for single cell into

a 96 well plate by a BD FACS Aria-II sorter. Single clones were verified by immunoblotting and sequencing using gene-specific primers:

ATL2-Fw: 5'-GTGAGAATTATGAAGATG-3',

ATL2-Rev: 5'-CTTGTTATACATGTATCTAAGC-3',

ATL3-Fw: 5'-ATTGGTTGGGTGACCCAGAAG-3',

ATL3-Rev: 5'-ACACAGTACATGCTGAAGTTC-3'.

Cells used in this study were not further authenticated after obtaining from the source. All cell lines were routinely tested and found negative for mycoplasma using a PCR-based method.

Immunofluorescence and confocal microscopy

For immunofluorescence experiments, cells were seeded on coverslips and transfected using Lipofectamine 3000 (Invitrogen) based on the manufacturer's instructions. 24 h after transfection, the cells were fixed in 4% paraformaldehyde (PFA) followed by permeabilization with 0.1% Triton X-100/PBS for 10 minutes. Cells were blocked in blocking solution (3% BSA in PBS) and incubated with anti-calreticulin (Abcam) and anti-HA (Zsbio) antibodies in blocking solution for 1 h at room temperature. Cells were washed in PBS, and then incubated with fluorophore-conjugated secondary antibodies (Thermo) for an additional 1 h. Cells were then washed and mounted on slides using mounting medium (KPL). Imaging was performed on a Zeiss LSM700 confocal microscope.

Quantification

ER three-way junctions were quantified in a 100 μm^2 region away from the nuclear. Quantification was performed using the Cell Counter function of Image J.

Supplementary References

1 Rismanchi, N., Soderblom, C., Stadler, J., Zhu, P. P. & Blackstone, C. Atlastin GTPases are required for Golgi apparatus and ER morphogenesis. *Hum Mol Genet* **17**, 1591-1604, doi:10.1093/hmg/ddn046 (2008).

2 Sun, S. *et al.* Identification of endoplasmic reticulum-shaping proteins in Plasmodium parasites. *Protein & cell* **7**, 615-620, doi:10.1007/s13238-016-0290-5 (2016).
