# Supplementary material for: Atlastin-1 regulates morphology and function of endoplasmic reticulum in dendrites
Source: Nat Commun. 2019 Feb 4;10:568. doi: 10.1038/s41467-019-08478-6 (PMC6362286; doi:10.1038/s41467-019-08478-6)
Supplement: Supplementary file 2 — Reporting Summary [file 41467_2019_8478_MOESM2_ESM.pdf]

nature research

Corresponding author(s): Xiangming Wang; Kang Shen

Reporting Summary

Nature Research wishes to improve the reproducibility of the work that we publish. This form provides structure for consistency and transparency in reporting. For further information on Nature Research policies, see Authors & Referees and the Editorial Policy Checklist.

Statistical parameters

When statistical analyses are reported, confirm that the following items are present in the relevant location (e.g. figure legend, table legend, main text, or Methods section).

n/a	Confirmed	
☐	☒	The exact sample size (*n*) for each experimental group/condition, given as a discrete number and unit of measurement
☐	☒	An indication of whether measurements were taken from distinct samples or whether the same sample was measured repeatedly
☐	☒	The statistical test(s) used AND whether they are one- or two-sided<br>*Only common tests should be described solely by name; describe more complex techniques in the Methods section.*
☒	☐	A description of all covariates tested
☒	☐	A description of any assumptions or corrections, such as tests of normality and adjustment for multiple comparisons
☐	☒	A full description of the statistics including central tendency (e.g. means) or other basic estimates (e.g. regression coefficient) AND variation (e.g. standard deviation) or associated estimates of uncertainty (e.g. confidence intervals)
☐	☒	For null hypothesis testing, the test statistic (e.g. *F*, *t*, *r*) with confidence intervals, effect sizes, degrees of freedom and *P* value noted<br>*Give P values as exact values whenever suitable.*
☒	☐	For Bayesian analysis, information on the choice of priors and Markov chain Monte Carlo settings
☒	☐	For hierarchical and complex designs, identification of the appropriate level for tests and full reporting of outcomes
☒	☐	Estimates of effect sizes (e.g. Cohen's *d*, Pearson's *r*), indicating how they were calculated
☐	☒	Clearly defined error bars<br>*State explicitly what error bars represent (e.g. SD, SE, CI)*

Our web collection on statistics for biologists may be useful.

Software and code

Policy information about availability of computer code

Data collection	Confocal images were acquired using Zeiss Axio Observer Z1 microscope （equipped with an Andor camera and a spinning-disk confocal scan head, FV10-ASW (version 2.0, for Olympus FV1200) and Zen (version 11.0.0.190, for Zeiss LSM700 and LSM880).<br>3D-SIM images were acquired on the DeltaVision OMX V3 imaging system (Applied Precision, GE). SIM image stacks were reconstructed using softWoRx (version 5.0, Applied Precision).<br>Some images in Figure S5 and S7 were acquired with Axio Imager M2 microscope (Carl Zeiss).
Data analysis	Microsoft Excel and GraphPad Prism (version: 5.0) were used for analyzing and graphing.

For manuscripts utilizing custom algorithms or software that are central to the research but not yet described in published literature, software must be made available to editors/reviewers upon request. We strongly encourage code deposition in a community repository (e.g. GitHub). See the Nature Research guidelines for submitting code & software for further information.

Data

Policy information about availability of data

All manuscripts must include a data availability statement. This statement should provide the following information, where applicable:

- Accession codes, unique identifiers, or web links for publicly available datasets
- A list of figures that have associated raw data
- A description of any restrictions on data availability

The quantification analyses in all figures have associated raw data. The raw data are available from the corresponding author upon reasonable request.

Field-specific reporting

Please select the best fit for your research. If you are not sure, read the appropriate sections before making your selection.

☒ Life sciences ☐ Behavioural & social sciences ☐ Ecological, evolutionary & environmental sciences

For a reference copy of the document with all sections, see nature.com/authors/policies/ReportingSummary-flat.pdf

Life sciences study design

All studies must disclose on these points even when the disclosure is negative.

Sample size	The sample size and the statistical method are indicated in the figure legends. There are no specific criteria for sample size in this study.
Data exclusions	No data were excluded from the analysis.
Replication	All attempts at replication were successful.
Randomization	This is not relevant to our study.
Blinding	The investigators were blinded to group allocations.

Reporting for specific materials, systems and methods

Materials & experimental systems

n/a	Involved in the study	
☒	☐	Unique biological materials
☐	☒	Antibodies
☐	☒	Eukaryotic cell lines
☒	☐	Palaeontology
☐	☒	Animals and other organisms
☒	☐	Human research participants

Methods

n/a	Involved in the study	
☒	☐	ChIP-seq
☒	☐	Flow cytometry
☒	☐	MRI-based neuroimaging

Antibodies

Antibodies used	Mouse Anti-HA mAb(Zsbio #TA-04),rabbit anti-calreticulin (Abcam #ab2907)
Validation	Mouse Anti-HA mAb(Zsbio #TA-04)<br>http://www.zsbio.com/product/TA-04<br>rabbit anti-calreticulin (Abcam #ab2907)<br>https://www.abcam.com/calreticulin-antibody-ab2907-references.html

Eukaryotic cell lines

Policy information about cell lines

Cell line source(s)	COS-7 cells were obtained from ATCC (Cat#ATCC CRL-1651, RRID: CVCL_0224).

Authentication	None of the cell lines were authenticated.
Mycoplasma contamination	All cell lines were tested negative for mycoplasma contamination.
Commonly misidentified lines<br>(See ICLAC register)	None

Animals and other organisms

Policy information about studies involving animals; ARRIVE guidelines recommended for reporting animal research

Laboratory animals	C. elegans
Wild animals	We do not use wild animals in our study.
Field-collected samples	We do not use Field-collected samples in our study.
